# The visuomotor transformations underlying target-directed behavior

**DOI:** 10.1073/pnas.2416215122

**Published:** 2025-03-24

**Authors:** Peixiong Zhao, Yuxin Tong, Ivan P. Lazarte, Biswadeep Khan, Guangnan Tian, Kenny K. Y. Chen, Thomas K. C. Lam, Yu Hu, Julie L. Semmelhack

**Affiliations:** ^a^Division of Life Science, The Hong Kong University of Science and Technology, Kowloon, Hong Kong Special Administrative Region, China; ^b^Department of Physiology, Anatomy and Genetics, University of Oxford, Oxford OX1 3PT, United Kingdom; ^c^Department of Chemical and Biological Engineering, The Hong Kong University of Science and Technology, Kowloon, Hong Kong Special Administrative Region, China; ^d^Department of Psychiatry and Behavioral Sciences, Stanford University, Stanford, CA 94305; ^e^Department of Computer Science and Engineering, The Hong Kong University of Science and Technology, Kowloon, Hong Kong Special Administrative Region, China; ^f^Neuroengineering Laboratory, Brain Mind Institute & Interfaculty Institute of Bioengineering, École Polytechnique Fédérale de Lausanne, Lausanne 1015, Switzerland; ^g^Department of Mathematics, The Hong Kong University of Science and Technology, Kowloon, Hong Kong Special Administrative Region, China

**Keywords:** visual, behavior, zebrafish, sensorimotor

## Abstract

The visual system can process diverse stimuli and make the decision to execute appropriate behaviors, but it remains unclear where and how this transformation takes place. Innate visually evoked behaviors such as hunting, freezing, and escape are thought to be deeply conserved, and have been described in a range of species from insects to humans. We found that zebrafish larvae would respond to predator-like visual stimuli with immobility and bradycardia, both hallmarks of freezing, in a head-fixed behavioral paradigm. We then imaged the zebrafish visual system while larvae responded to different visual stimuli with hunting, freezing, and escape behaviors and systematically identified visually driven neurons and behaviorally correlated sensorimotor neurons. Our analyses indicate that within the optic tectum, broadly tuned sensory neurons are functionally correlated with sensorimotor neurons which respond specifically during one behavior, indicating that it contains suitable information for sensorimotor transformation. We also identified sensorimotor neurons in four other areas downstream of the tectum, and these neurons are also specific for one behavior, indicating that the segregation of the pathways continues in other areas. While our findings shed light on how sensorimotor neurons may integrate visual inputs, further investigation will be required to determine how sensorimotor neurons in different regions interact and where the decision to behave is made.

## Significance

Freezing is an innate fear response triggered by visual stimuli. Here, we developed a visually evoked freezing paradigm in larval zebrafish and showed that presenting a moving dark disk could trigger immobility, as well as bradycardia, another hallmark of freezing. We combined this assay with escape and hunting paradigms and large-scale imaging. We found that sensory neurons for the stimuli were located primarily in the optic tectum, while behavior-correlated sensorimotor neurons for the three behaviors were located in the tectum as well as three other areas, suggesting that the tectum contains both sensory and sensorimotor information. However, whether the decision to behave is made in the tectum or via another area remains to be determined.

Animals use their visual system to detect an array of visual targets and respond with appropriate behaviors. Prey stimuli trigger orientation, approach, and consumption behaviors (1–3), while predator-like stimuli can evoke fighting, fleeing, or freezing (4, 5). Fleeing involves rapid movement, which may be directed away from the potential threat (6–9). Freezing, on the other hand, is a cessation of movement, which may help the animal avoid detection, accompanied by a decrease in heart rate or bradycardia (10–13).

In many systems, these behaviors can be evoked by simple visual stimuli; small moving objects represent prey and trigger hunting behavior (14, 15), while looming stimuli can represent an approaching predator and trigger escape (7–9, 16). In contrast, an object sweeping across the visual field without changing size has been shown to evoke freezing in both flies and mice (17–19), and this stimulus may mimic a predator that is cruising but not approaching. The detection of these stimuli within the early visual system is partially understood (7, 8, 15, 20, 21), but it remains unclear, particularly in vertebrates, how the visual information is transformed into the appropriate behavioral output.

In order to perform these visual behaviors, the animal must 1) detect the identity and position of the stimulus, 2) decide whether and how to respond, and 3) execute a directed motor program toward or away from the target (in the cases of hunting and escape). The decision of whether to respond and with which behavior may be dependent on satiety, context, locomotor state, or other kinds of sensory input (22–25). For target-directed behaviors, which are triggered by visual stimuli located at a particular point in space, the optic tectum (superior colliculus in mammals) is a likely site for visuomotor transformation, as it receives retinal input, contains a map of visual space, has outputs to many motor areas, and receives a variety of other sensory and state inputs (26–28). However, other brain areas have also been implicated in these behaviors (29–31), and their manipulation has been shown to affect hunting and defensive behaviors (32–34). In addition, it has been difficult to comprehensively record from these areas, particularly during behavior, in order to observe how the animal decides whether to respond and selects the appropriate type of behavior.

Here, we first characterize visually evoked freezing (immobility and bradycardia) in response to a sweeping stimulus in zebrafish larvae. In addition to flies (23), rodents (17), and humans (11), freezing behavior has also been found in zebrafish; adult zebrafish exhibit immobility in response to threatening stimuli such as novel environments or alarm pheromone (35, 36), and late-stage larvae have a bradycardia response during conditioned fear (12, 37). However, visually evoked freezing had not been described in zebrafish. To investigate the sensorimotor circuits mediating this behavior and how they relate to the pathways for other visual behaviors, we presented the sweeping stimulus as well as prey and looming stimuli and observed whether the larvae responded by freezing, hunting, or escaping. We used volumetric 2p calcium imaging to map the sensory neurons that respond to predator-like and prey stimuli and the sensorimotor neurons that are correlated with stimulus-evoked freezing, escape, and hunting. We identified sensory neurons for the stimuli in the tectum and found that sensorimotor neurons were also located there, as well as in several other downstream areas. Our data suggest that the sensorimotor neurons in the tectum integrate visual input from sensory neurons and may comprise a key node in the pathways that convert sensory input into different behavioral outcomes.

## Results

### A Large Translating Disk Causes Immobility.

**Fig. 1.**
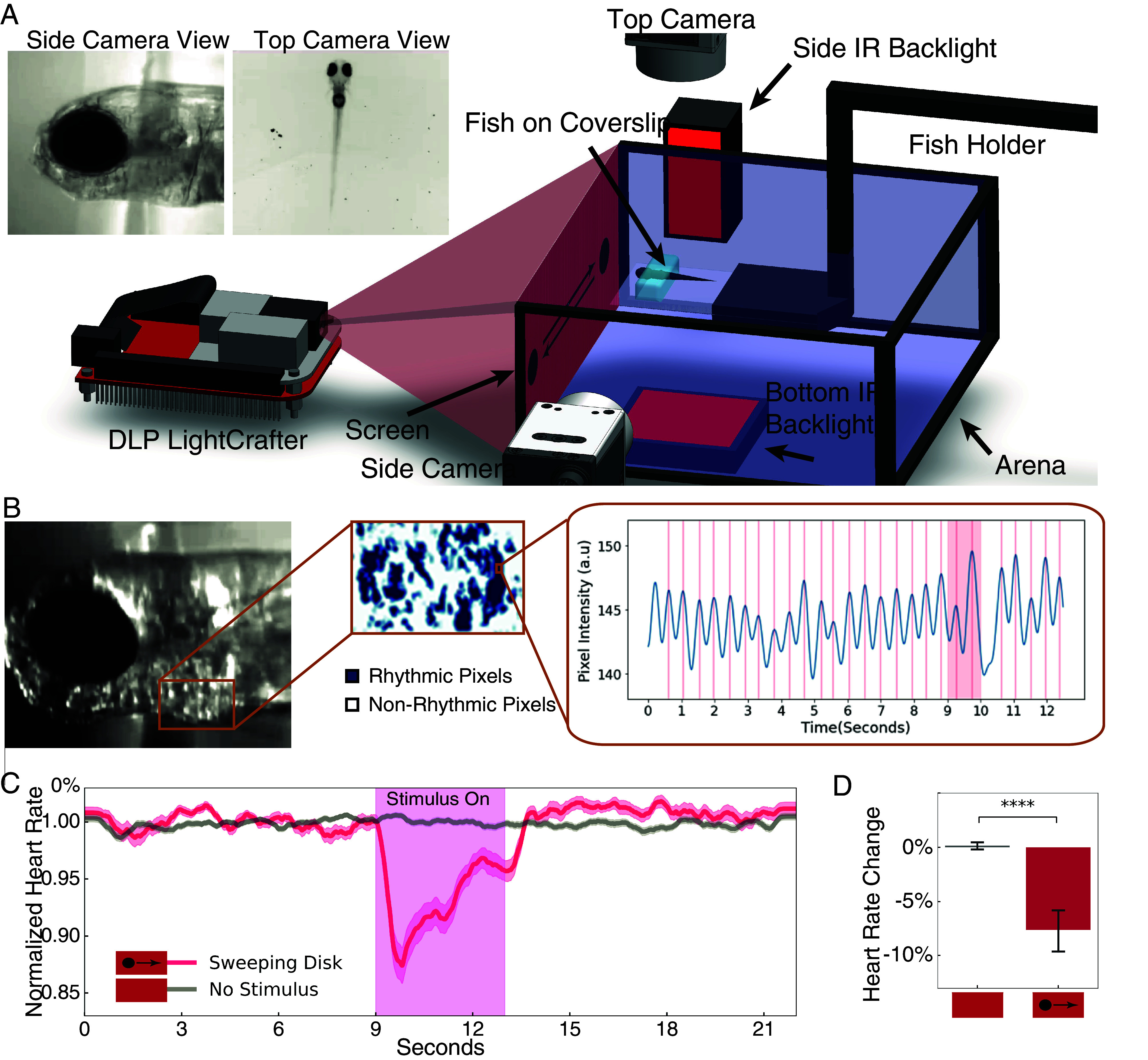
A large translating disk causes immobility and bradycardia. (*A*) Schematic of the experimental setup. (*B*) Identification of rhythmic pixels within the heart ROI and pixel intensity peaks corresponding to heart beats. (*C*) Normalized heart rate in response to sweep or no stimulus. Shading indicates SE. (*D*) Change in heart rate during the 3 s after stimulus onset. n = 14 larvae. Error bar = SD. ****, *P* < 0.0001, Mann–Whitney U test.

To test whether a visually evoked freezing response could be triggered in zebrafish larvae, we designed a 15° disk stimulus that would sweep horizontally across the frontal visual field. This initial stimulus was similar in size to that used to trigger freezing in other systems (17, 19) and larger than a prey stimulus (15). We projected the dark sweeping disk on a red background onto the screen of our behavior chamber (Fig. 1*A*). Within the chamber, a head-fixed 6- or 7-d postfertilization larva was mounted 1 cm from the screen, and we used high-speed cameras above and to the side to monitor its behavior (Fig. 1*A*, *Insets*). The larvae would intermittently perform spontaneous swims, and we found that the presentation of the sweeping stimulus would suppress swimming for several seconds (Movie S1). When we quantified swim probability (probability of a swim bout occurring in that frame) across all sweeping stimulus trials from several larvae, we found that there was a decrease in swim probability from stimulus onset for a period of about 10 s (*SI Appendix*, Fig. S1*A*, *Upper* panel), This decrease can be quantified as a change in swim probability that was significantly greater for the stimulus vs. no stimulus trials (*SI Appendix*, Fig. S1*B*). This suggests that the sweeping stimulus was suppressing spontaneous swims and causing immobility, one of the signatures of freezing.

### Bradycardia Can Be Used as an Indicator of Freezing.

In many species, freezing is accompanied by a decrease in heart rate that occurs at the onset of immobility (10, 11,38). Therefore, we asked whether our putative freezing stimulus would also cause bradycardia, in addition to suppressing movement. We used the side camera to record the heart beating and analyzed heart rate by measuring the change in intensity of rhythmically active pixels (*Methods*) (13). We calculated the heart rate for each frame of the video by finding the distance between local peaks in the intensity curve of the rhythmically active pixels (Fig. 1*B*). We found that the dark sweeping disk robustly triggered a reduction in heart rate at the onset of stimulus presentation (Fig. 1*C* and Movie S2), and the heart rate change during the stimulus time window was significantly greater than with no stimulus (Fig. 1*D*). We next asked whether bradycardia and the decrease in movement were linked and defined bradycardia as a decrease in heart rate to ceiling greater three SD from the mean (*Methods*) starting during the stimulus presentation. We found that bradycardia was associated with a decrease in swimming probability during and for several seconds after the stimulus (*SI Appendix*, Fig. S1*A*, *Lower* panel), and trials with bradycardia had significantly less movement than those without (*SI Appendix*, Fig. S1*C*). Taken together, these data suggest that as in other systems, a dark sweeping visual stimulus of constant size triggers the innate freezing response to potential predators, which can be observed as a cessation of movement and transient decrease in heart rate. As spontaneous swim probability is quite variable between larvae, we used bradycardia as the behavioral readout for subsequent experiments.

### Optimal Size and Speed Parameters to Induce Bradycardia.

We next explored the stimulus parameters to identify those that would most effectively induce bradycardia. We presented dark sweeping disks on red, UV, and green backgrounds and found that all were effective at triggering bradycardia (*SI Appendix*, Fig. S1 *D* and *E*). In comparison, a bright disk on a dark background was less effective than the dark stimuli (*SI Appendix*, Fig. S1 *D*–*G*). We therefore chose to use the dark disk on a red background for further experiments. Next, we varied stimulus speed and found that a 15° diameter disk with a broad range of speeds triggered bradycardia (*SI Appendix*, Fig. S1*H*), and we chose 60°/s as a value in the middle of that range. Using a speed of 60°/s, stimuli of 5 to 30° degrees in diameter strongly reduced the heart rate (*SI Appendix*, Fig. S1*I*), and we thus used a 15° diameter, 60°/s disk for further experiments.

### Functional Imaging to Identify Sensory Neurons That Respond to Each Stimulus.

**Fig. 2.**
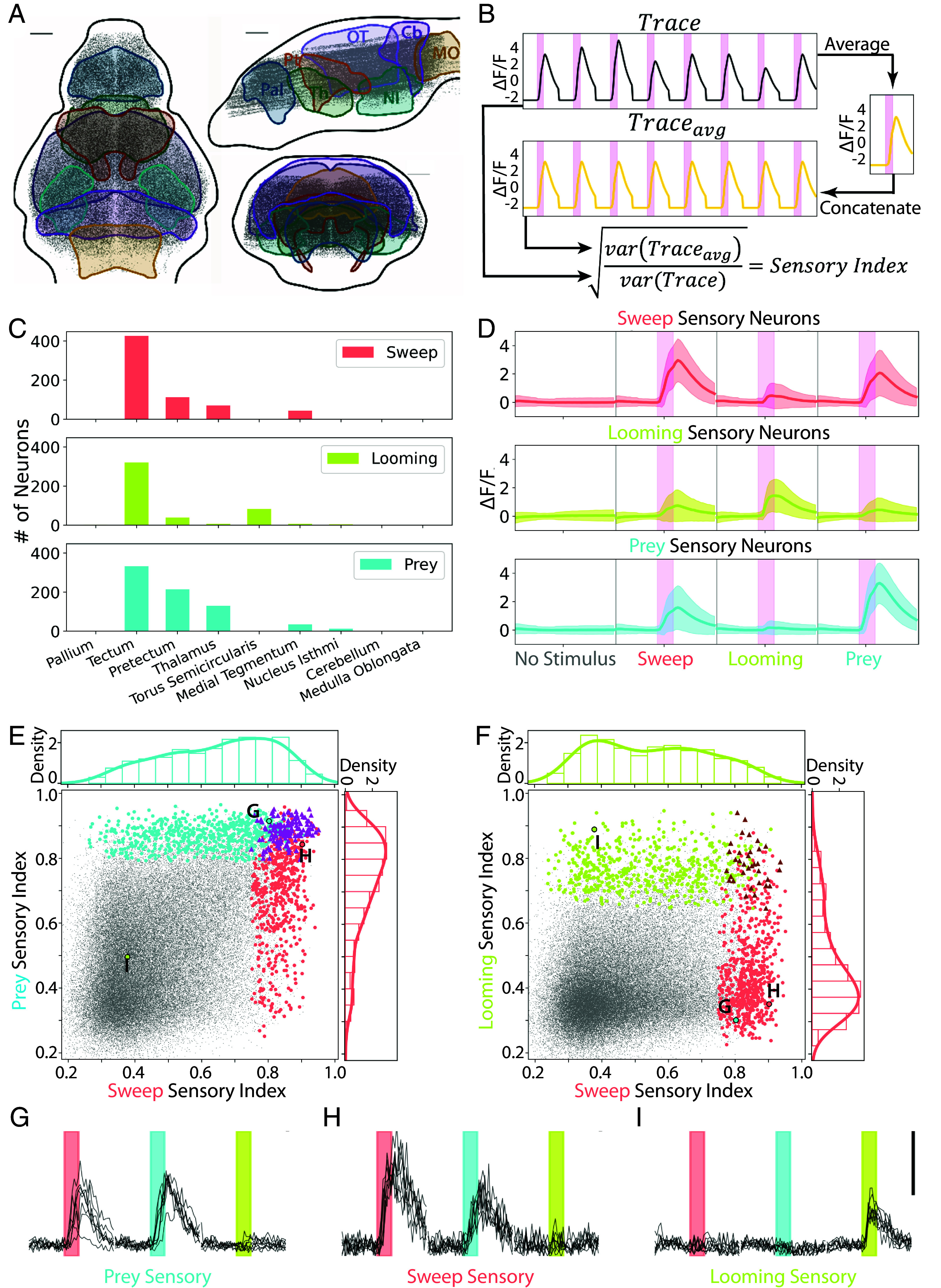
Identification of sensory neurons for sweep, prey, and looming stimuli. (*A*) Cell bodies in the imaging dataset (n = 7 larvae, 188,272 neurons). Pallium, Pal; Pretectum, Pt; Thalamus, Th; Optic Tectum, OT; Nucleus Isthmi, NI; Cerebellum, Cb; Medulla Oblongata, MO. (Scale bar, 50 µm.) (*B*) Calculation of the sensory index (SI) of each neuron. (*C*) Number of sensory neurons in each brain area. (*D*) The average response of each sensory neuron population to all stimuli. Pink bars = 4 s stimulus display. Shading indicates SD. (*E*) SI of all sweep (red), prey (blue), and prey + sweep (purple) sensory neurons, and all active neurons in the dataset (gray, 63,420 neurons). Density plots represent sensory neurons of each type. (*F*) SI of looming (green), sweep (red), and looming + sweep (brown) sensory neurons. (*G*–*I*) Response of example sweep, prey, and looming sensory neurons selected near the mode (i.e., max density) of that population’s density plot. Red, blue, and green bars indicate 4 s sweep, prey, or looming stimuli. Scalebar indicates ΔF/F = 3.

To investigate the freezing pathway and how it compares to those for hunting and escape, we adapted our freezing behavioral assay for volumetric 2p calcium imaging and added prey and looming stimuli. The prey stimulus, was a small (4° diameter) sweeping UV dot with a similar horizontal trajectory and speed as the sweep stimulus, which triggers hunting behavior (*SI Appendix*, Fig. S2*A* and Movie S3) (39, 40), as defined by eye convergence and prey capture swims (15, 41). The looming stimulus is a dark disk that expands from 6 to 60° in diameter (*SI Appendix*, Fig. S2*A*). This stimulus primarily evokes escape (Movie S4), which involves a high velocity and amplitude turn away from the stimulus (7, 8). We presented each stimulus eight times, with a 2 min interstimulus interval. We used larvae expressing nuclear localized GCaMP6s pan-neuronally (*elavl3:Hsa.H2B-GCaMP6s* (42), aka *elavl3*) and an electrically tunable lens to image a large volume of the brain, including many of the areas thought to be involved in defensive behaviors (Fig. 2*A*). We recorded from 14 planes at 2 Hz, covering about 2/3 of the brain by volume (Fig. 2*A* and *SI Appendix*, Table S1). We used suite2p (43) to motion correct and segment cell bodies, identifying a total of 188,272 cell bodies within our dataset of 7 fish, and mapped the cell bodies onto the mapZebrain atlas (44). Within the total population of segmented cell bodies, we then defined a population of active neurons (correlated with one of the stimuli or behaviors in 30% of the relevant trials (*Methods*): 63,420 neurons in total) to streamline the subsequent analysis.

We first identified the purely stimulus-correlated neurons, or “sensory neurons” for each stimulus. We adopted an approach similar to a recent study (45), based on the idea that sensory neurons should respond to repeated presentation of the same stimulus with high periodicity. For each neuron, we calculated its average response to a given stimulus over eight repeats and generated the average trace (Trace_avg_) by concatenating the average response. We then calculated a Sensory Index (SI) for the neuron’s response to that stimulus, which reflects the variance explained by the periodic component of the response (*Methods*), such that if the average and actual trace have the same variance, the SI will have the maximum value (Fig. 2*B*). Neurons with an SI in the first percentile of the population had high periodicity, responding every time the stimulus was presented, while neurons with lower SI were less regular in their responses (*SI Appendix*, Fig. S2*B*).

Use of periodicity alone to identify sensory neurons gave us a significant proportion of neurons with flat or slowly ramping traces. Therefore, we first selected neurons that were correlated with the stimulus regressor (top 10%, see *Methods*), then within that population took the top 15% based on the SI. Neurons at the lower limit of this range still responded regularly to the stimulus (*SI Appendix*, Fig. S2*B*, *Middle* trace), so we chose 15% as a threshold for sensory neuron identification. We further filtered the population for neurons that were colocalized across multiple animals (spatial colocalization test, *Methods*) (46). Within our population of active neurons, we identified 682 sweep, 556 looming, and 746 prey sensory neurons in total. We found that the vast majority of the sensory neurons for all three stimuli were in the optic tectum, while the prey stimulus also activated a large population within the pretectum (Fig. 2*C*), consistent with previous studies (15, 47). To validate our selection of 15% of the SI as a threshold, we compared the anatomical distribution of sensory neurons for thresholds ranging from 5% to 30% SI and found that the fraction of neurons in each brain area was consistent (*SI Appendix*, Fig. S2 *C–E*). We also plotted the proportion of sensory neurons from each larva and found that these reliably stimulus-driven neurons in the tectum were present in all seven fish (*SI Appendix*, Fig. S2*F*).

We next examined the functional properties of the three populations of sensory neurons and found that sweep sensory neurons on average responded robustly to the sweeping disk, as expected, and also to the prey dot (Fig. 2*D*, red). Looming sensory neurons responded primarily to the looming stimulus (Fig. 2*D*, green), while prey sensory neurons on average responded to the sweeping disk and the prey dot (Fig. 2*D*, blue). To further examine the responses of individual neurons, we use a scatter plot to visualize each neuron’s sweep sensory index (SI) vs. its prey SI. As expected, most sweep sensory neurons had a high sweep SI (Fig. 2*E*, red dots), and a large proportion of them also had a prey SI of ~0.8 (Fig. 2*E*, red density plot). The response of an example neuron from the mode of the sweep sensory neuron distribution shows a fairly periodic response to the prey stimulus (Fig. 2*H*). Similarly, the prey sensory neurons (Fig. 2*E*, blue) all had a high prey SI, and many of them also had a sweep SI of ~0.8 (Fig. 2*E*, blue density plot), indicating regular activation by the sweep stimulus (Fig. 2*G*). There was also a significant population of neurons that were classified as both sweep and prey sensory neurons (Fig. 2*E*, purple triangles). In contrast, most sweep sensory neurons had a relatively low looming SI of ~0.4 (Fig. 2*F*, red density plot, and 2H). Looming sensory neurons similarly had low SI for sweep (Fig. 2*F*, light green density plot, and Fig. 2*I*). These results suggest that the tectal sensory neurons may be encoding object location more specifically than object identity (i.e. prey vs. predator), as there was substantial overlap between the stimuli with similar trajectories (sweep and prey), but not between the two dark, predator-like stimuli (sweep and looming).

### Identification of Sensorimotor Neurons Correlated With Each Behavior.

**Fig. 3.**
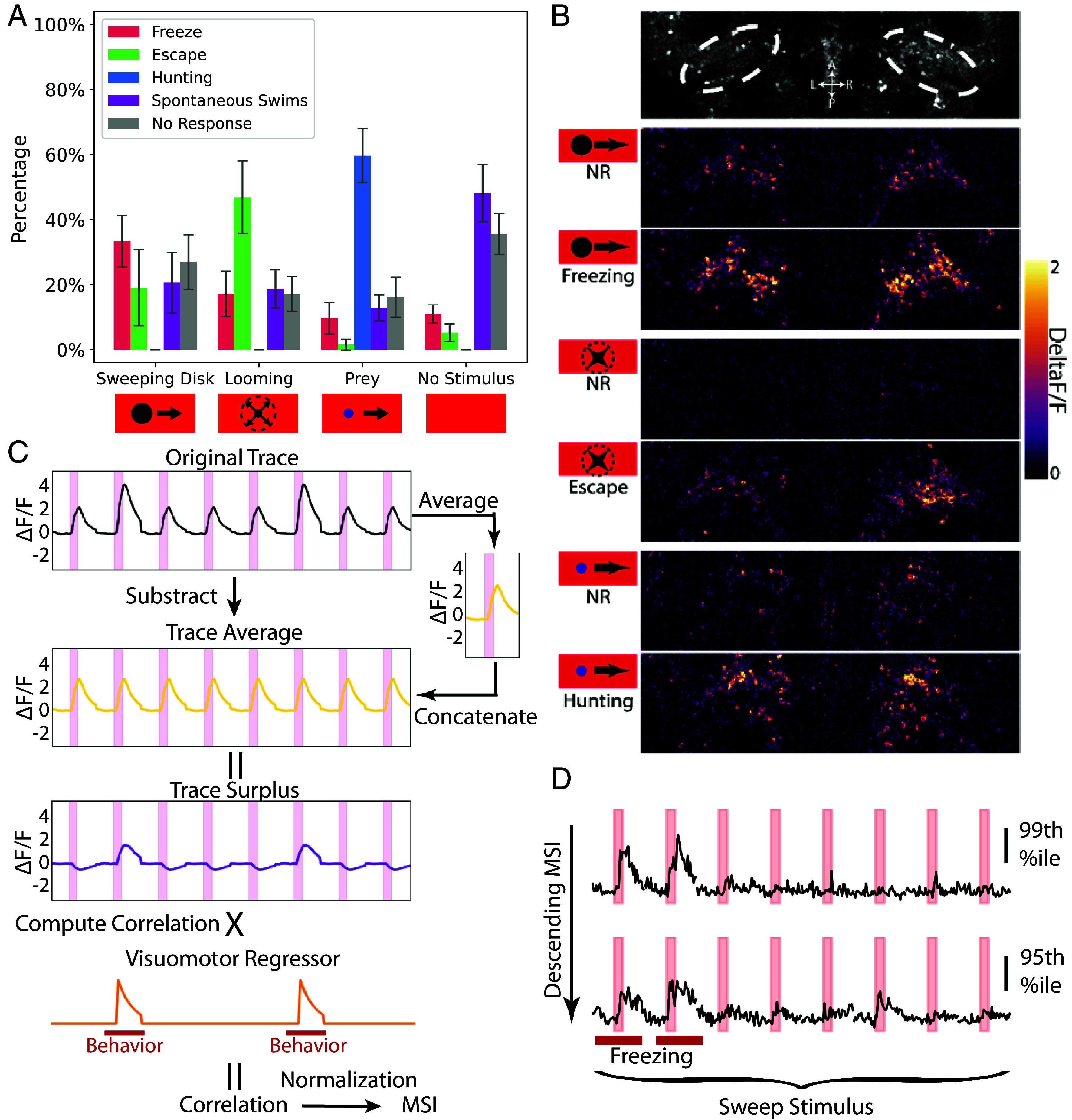
Identification of sensorimotor (SM) neurons for freezing, escape, and hunting. (*A*) Percent of trials with each behavioral response to the stimuli. The error bar represents SD. n = 7 larvae (*B*) Example responses of cell bodies in the NI to sweep, looming, and prey stimuli in no behavioral response (NR) and freezing, escape, and hunting trials. (*C*) Schematic calculation of freezing MSI for a neuron in a fish with freezing in the second and 6th trials. The trace average is subtracted from the original trace to generate the trace surplus, and then, the Pearson correlation between the trace surplus and the visuomotor regressor is computed to give the freezing motor surplus index (MSI) for that neuron. (*D*) Example ΔF/F neuronal traces during the eight sweep presentations from a larva with two freezing trials. Neurons are from the 99th and 95th percentile of the freezing MSI. Red bars indicate freezing trials.

Given that animals do not respond with the identical behavior to each repeat of a visual stimulus, at some point within the visuomotor pathway, visual information must be integrated with state, history, or other sensory information to make the decision to behave. The neurons at this point and later in the pathway can be thought of as “sensorimotor” neurons (SM neurons) and will be correlated with a behavior which is evoked by a particular visual stimulus. To search for these neurons in our imaging dataset, we first classified the behaviors triggered by the three stimuli as hunting, freezing, and escapes. Trials containing eye convergence bouts during stimulus presentation were classified as hunting, those with fast, large amplitude swims were classified as escape, those with no movement and bradycardia were classified as freezing, and those with multiple types of behavior during the stimulus window were excluded from the analysis (*SI Appendix*, Fig. S3 *A* and *B*). Based on this classification, the sweeping stimulus triggered mostly freezing and some escape, while the looming stimulus largely evoked escape and a smaller percentage of freezing trials (Fig. 3*A*).

Within certain brain areas, we observed that some neurons had little response to our stimuli in trials without behavior, but were robustly activated in behavior trials, with different sets of neurons responding during the three behaviors (NI, Fig. 3*B*). To systematically identify these SM neurons, inspired by the same prior study (45), we designed an analysis method that would decompose neuronal responses into stimulus-dependent and -independent components. For each neuron, we calculated a “trace surplus,” or stimulus-independent trace, by subtracting the average response to a given sensory stimulus. Subtracting the trace average, or the neuron’s typical response to the visual stimulus, allowed us to isolate the behavior-related component of each neuron’s activity (the trace surplus, Fig. 3*C*) and more clearly differentiate sensory and SM neurons than would be possible with a simple regression analysis. We then calculated the Pearson correlation between the trace surplus and a visuomotor regressor (*Methods* and *SI Appendix*, Fig. S4) and used that correlation score to calculate a MSI for the primary behavior driven by each stimulus.

**Fig. 4.**
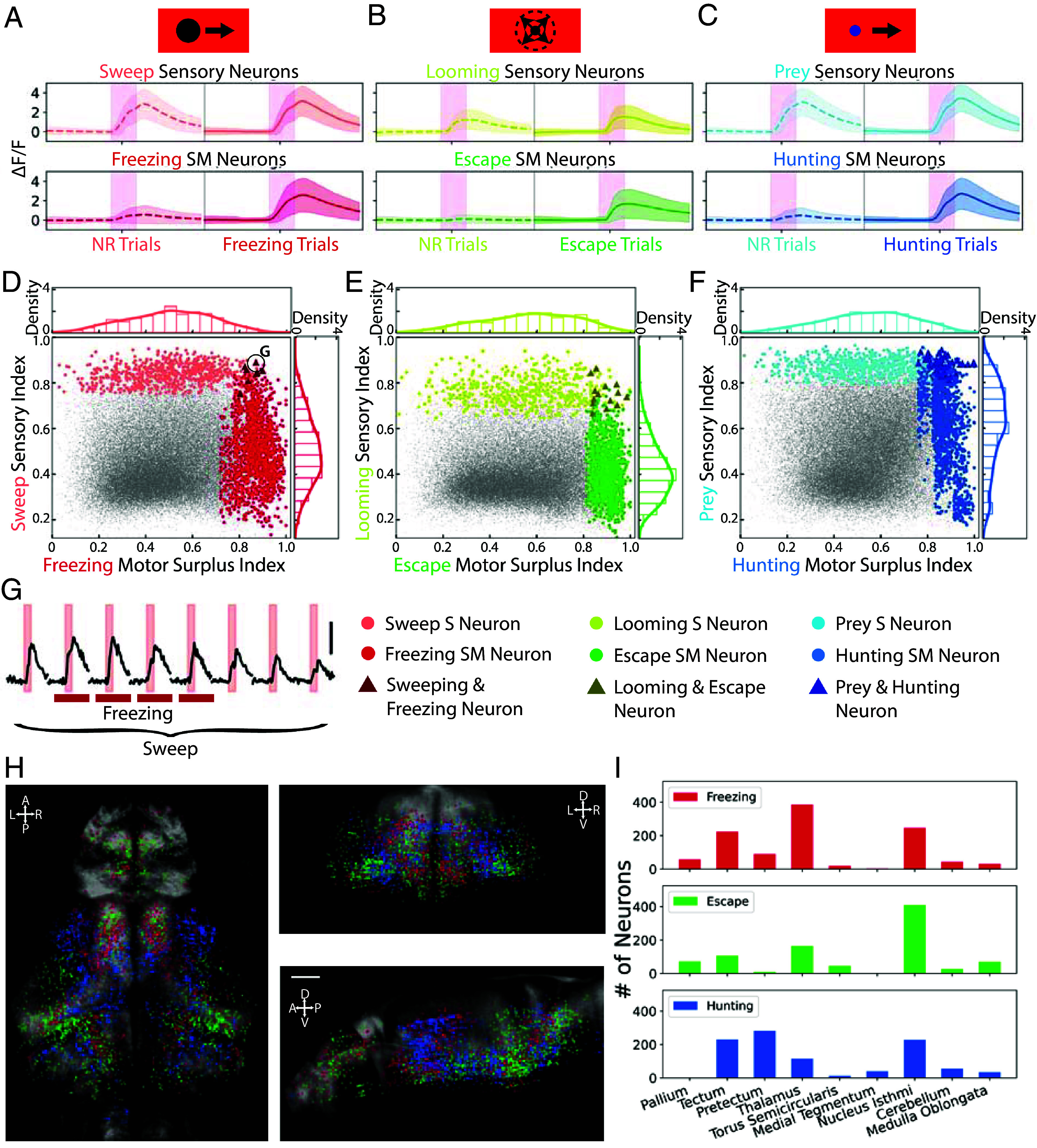
Functional properties and anatomical locations of sensorimotor (SM) neurons for freezing, escape, and hunting. (*A*–*C*) Average calcium response of the sensory and SM neurons during behavior and no response trials. (*D*) The distribution of sweep sensory neurons (light red) and freezing SM neurons (dark red) on the sweep SI and freezing MSI. Crimson triangles: neurons belonging to both populations. (*E*) The distribution of the looming sensory neurons (light green) and escape SM neurons (dark green) on the looming SI and escape MSI. Gray triangles: neurons belonging to both populations. (*F*) The distribution of the prey sensory neurons (light blue) and hunting SM neurons (dark blue) on the prey SI and hunting MSI. Navy triangles: neurons belonging to both populations. (*G*) Response of example neuron with both high sweeping SI and freezing MSI, annotated in *D*. Light red bars: 4 s presentation of sweep stimuli. Dark red bars: trials with a freezing response. The scale bar indicates ΔF/F = 3. (*H*) Locations of SM neurons in the recording volume. Red: Freezing neurons. Green: Escape neurons. Blue: Hunting neurons. (Scale bar, 50 µm.) (*I*) Numbers of SM neurons in each brain area.

As expected, neurons with a high MSI responded more robustly in behavior trials, whereas lower-scoring neurons were less correlated with behavior (Fig. 3*D*). To determine an appropriate MSI threshold, we calculated the motor enhancement, or difference between no response and behavior trials of the sensorimotor neuron populations (*SI Appendix*, Fig. S5*A*). Motor enhancement was greatest in the 99th percentile neurons by MSI and lower but still present in the 90th percentile (*SI Appendix*, Fig. S5*B*). We found an elbow in the degree of motor enhancement around the 97th percentile for all behaviors (*SI Appendix*, Fig. S5*C*. We thus defined the 97th percentile of MSI as the threshold for the three types of SM neurons. After spatial colocalization filtering, we were left with 1,219 freezing, 1,239 escape, and 1,262 hunting SM neurons in total. Using this approach, we were able to identify populations of SM neurons that had a more robust response during behavior trials, in contrast to the sensory neurons (Fig. 4*A*–*C*).

To look more closely at the response properties of individual neurons within the SM populations, we plotted SI vs. MSI of each stimulus and behavior pair. We found that the freezing SM neurons (Fig. 4*D*, dark red) typically have a low sweep SI, with the mode of the distribution around 0.4, indicating little periodicity for the sweep stimulus. Although it is possible for neurons to be classified as both sweep sensory and freezing SM neurons (maroon triangles), there were only eight neurons of this type in our entire imaging volume (Fig. 4*D* and example trace, Fig. 4*G*). The counts of looming + escape (Fig. 4*E*) and prey + hunting populations (Fig. 4*F*) were similarly low. Thus, this analysis allows us to identify behavior-correlated neurons that are almost completely distinct from the sensory populations.

We next looked at the anatomical locations of each SM neuron type within the mapZebrain atlas (44). SM neurons for the three behaviors were distributed in distinct patterns within our imaging volume (Fig. 4*H*). There was a significant population of hunting, freezing, and escape SM neurons in the tectum, as well as in the thalamus and NI (Fig. 4*I*). A large population of hunting SM neurons was also found in the pretectum (Fig. 4*I*), consistent with previous findings (32). The anatomical locations of SM neurons were fairly consistent across fish (*SI Appendix*, Fig. S5*D*). We also compared the locations of the top 600 neurons in each fish by MSI prior to within fish normalization and the spatial colocalization test and found that the patterns and brain regions were still similar across fish (*SI Appendix*, Figs. S6–S8), although there were subtle differences, for example in the number of freezing SM neurons in the thalamus. This may be due to differing numbers of behavior trials between fish; in a fish with several freezing trials (e.g. fish 6, *SI Appendix*, Fig. S6*A*), many neurons in the thalamus have a high MSI, and these will take up a larger share of the top 600. A fish with only one freezing trial, e.g. fish 1, will have more randomly distributed neurons, which will be eliminated by the spatial colocalization test.

To validate our method of selecting SM neurons, we next asked whether the activity of an SM population can predict behavior. We conducted a separate selection of freezing SM neurons, using the freezing annotation and neuronal activity of seven trials, with one trial held out (*SI Appendix*, Fig. S9*A*). We then compared the held-out trial activity to the maximum population activity during the freezing and no response trials (*SI Appendix*, Fig. S9*B*) to ask whether they could predict whether the held-out trial was a freezing or no response trial. We also asked how well a set of null SM neurons, selected based on a permuted behavioral annotation (*SI Appendix*, Fig. S9*C*) could predict the permuted annotation. We found that the real freezing SM neurons were significantly better than the null population at predicting whether the larva froze in the held-out trial, with an accuracy of 84.8% (*SI Appendix*, Figs. 9*D* and 10).

### Anatomy and Function of Tectal Sensorimotor Neurons.

As the tectum was the area with by far the largest concentration of sensory neurons of the three types (Fig. 2*C*), and it also contains SM neurons for the three behaviors, it seems likely that the sensory neurons could provide input to the corresponding population of SM neurons. We therefore looked at the anatomical distributions of SM neurons for clues as to how such interactions could occur.

**Fig. 5.**
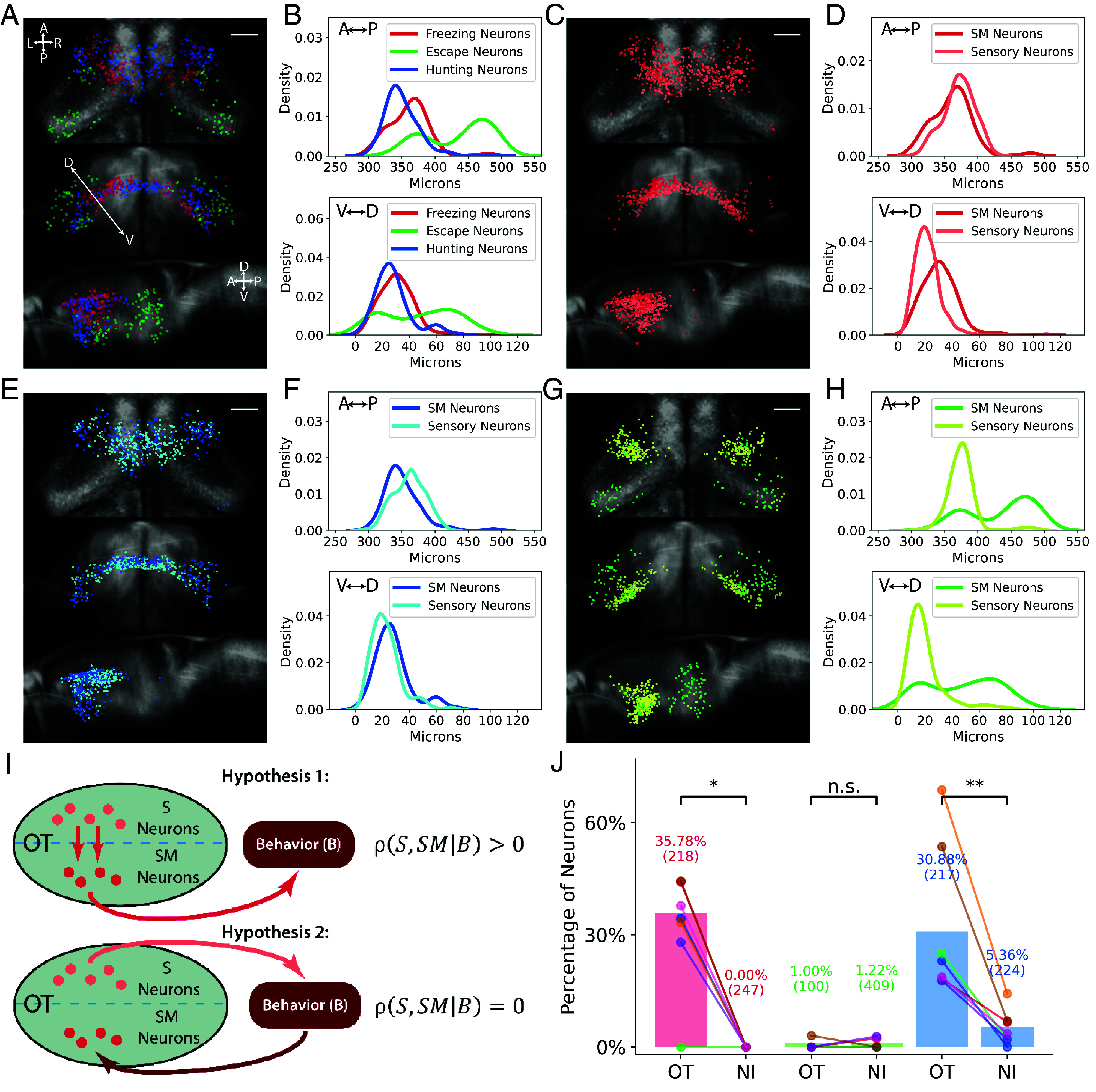
Sensory and sensorimotor neurons in the tectum. (*A*) Locations of the three types of SM neurons in the tectum. (Scale bar, 50 µm.) (*B*) Locations of SM neurons on the anterior/posterior and dorsal/ventral axes. (*C* and *D*) Locations of sweep sensory neurons (light red) and freezing SM neurons (dark red) in the tectum and their locations on the anterior/posterior and dorsal/ventral axes. (*E* and *F*) Locations of prey sensory neurons (light blue) and hunting SM neurons (dark blue) in the tectum and on the anterior/posterior and dorsal/ventral axes. (*G* and *H*) Locations of looming sensory neurons (light green) and escape SM neurons (green) in the tectum and their locations on the anterior/posterior and dorsal/ventral axes. (*I*) Hypotheses for the tectum S and SM connectivity in the sensorimotor circuit. (*J*) Percentage of SM neurons that were significantly correlated with the tectal sensory neurons. Colored dots represent values for individual fish. Parenthesis: total number of SM neurons of that type across all fish. NI = Percentage of SM neurons in the NI that were significantly correlated with tectal sensory neurons. Percentage-by-chance was 0% for both tectal and NI SM neurons (*Methods*). *, *P* < 0.05. **, *P* < 0.01, one-sided Wilcoxon signed-rank test. The *P*-values were 0.011 for freezing, 0.787 for escape, and 0.007 for hunting.

Hunting, freezing, and escape SM neurons were located in distinct regions of the tectum (Fig. 5*A*), with hunting neurons in the most anterior position, freezing neurons in the middle, and escape neurons most posterior (Fig. 5*B*), consistent with previous results (48, 49). To ask whether optogenetic stimulation of different regions of the tectum could produce our behaviors, we expressed ChR2 under control of *Gal4s:1013t* (50) and used an optic fiber to activate the anterior vs. posterior tectum. We observed significantly more eye convergence bouts when we stimulated the anterior vs. posterior tectum, while stimulation of the posterior tectum produced more escape bouts (*SI Appendix*, Fig. S11), suggesting that hunting and escape SM neurons in the anterior and posterior regions of the tectum, respectively, could mediate hunting and escape responses.

We next looked at where each set of sensory neurons was located relative to its sensorimotor pair. The sweep sensory and freezing SM neurons were located in similar regions of the tectum along the anterior/posterior axis (Fig. 5*C* and *D*), with the SM neurons slightly dorsal to the sensory neurons. The prey sensory and hunting SM neurons were also located in similar regions, with the sensory population slightly more posterior (Fig. 5*E* and *F*). In contrast, the anatomical separation between the looming sensory and escape SM neurons was more pronounced (Fig. 5*G* and *H*). These data suggest that the sensory and SM neurons of each pair constitute separate anatomical populations, which may be connected to each other.

### Using Partial Correlation Analysis to Assess Connectivity Within the Tectum.

To ask whether sensory and SM neurons in the tectum are connected, we looked for correlations between the activities of sensory and SM neurons of each type. If sweep sensory neurons are connected to freezing SM neurons, we would expect the two populations to be correlated (Fig. 5*I*, hypothesis 1). However, it is also possible that the freezing SM neurons in the tectum are not receiving input from their sensory counterparts but are nonetheless correlated to them due to indirect pathways via some other brain area that determines behavior (hypothesis 2). To distinguish the two scenarios, we adapted statistical methods of testing different dependency hypotheses (51), which are based on the partial correlation between the sensory and SM neurons while conditioning on the behavior $\rho \left(S,SM|B\right)$ (Fig. 5*I*). Intuitively, only in hypothesis 1 are the sensory and SM neurons still correlated once their relationship with the behavior is removed.

To remove the behavior-related activity, we calculated a residual activity trace for each SM neuron, analogous to the trace surplus in Fig. 3*C*. First, we compute behavior and nonbehavior averages (*SI Appendix*, Fig. S12*A*, *Middle* row). Both templates are then subtracted from the original trace to obtain the residual trace (*SI Appendix*, Fig. S12*A*, *Bottom* row). The subtraction of the behavioral and nonbehavioral averages ensures that correlations are not due to input from a separate area which is actually making the behavioral decision (hypothesis 2, Fig. 5*I*). The sensory neurons’ residuals are calculated in the same way. The partial correlation given behavior $\rho \left(S,SM|B\right)$ is then calculated as the Pearson correlation coefficient between the SM and sensory residual. Next, we determined whether such partial are significant. If the S and SM neurons are connected, each SM neuron probably receives inputs from only a subset of sensory neurons. Therefore, for each SM neuron, we quantify its strongest correlations by calculating the 90th percentile of its partial correlations with all sweep sensory neurons (*SI Appendix*, Fig. S12*C*, *Right*). To check for significance, we compare this 90th percentile against a null distribution using trial-shuffled data (permuted trace, *SI Appendix*, Fig. S12*B*), to obtain an empirical p-value (*SI Appendix*, Fig. S12 *C* and *D*).

After adjustment for multiplicity, we found that substantial fractions, 35.78 and 30.88%, of the freezing and hunting SM neurons have a significant partial correlation with corresponding sensory neurons (Fig. 5*J*). For escape SM neurons, we only observed a small fraction (1.00%) with a significant partial correlation with looming sensory neurons. According to partial correlation theory (51), these results argue against hypothesis 2 and support hypothesis 1, sensory and SM neurons are connected, at least for freezing and hunting behaviors. One caveat is that our analysis relies on partial correlations; thus, validation of causal effects in the proposed circuit (hypothesis 1), for example through perturbation experiments, is needed in future work.

To further verify the method, we applied the analysis to a number of controls. First, if the tectal sensory neurons are uniquely providing input to tectal SM neurons, SM neurons in other areas such as the NI should not be as correlated with tectal sensory neurons. Using the same analysis pipeline, we found 0 and 5.36% of NI SM neurons for freezing and hunting were significantly correlated with tectal sensory neurons (Fig. 5*J*). Second, we verified that correlations between residuals are due to within-trial fluctuations in activity rather than the subtraction of the average traces (*SI Appendix*, Fig. S13). Third, we confirm that the observed partial correlation between tectal SM and sensory neurons is not due to artifacts related to two-photon scanning or imaging noise (*SI Appendix*, Fig. S14).

It is also possible that sensorimotor transformations could occur in the pretectum and thalamus, as these areas also contain sensory and SM neurons. We performed the partial correlation analysis for freezing and hunting behaviors in these two areas (escape could not be analyzed because only eight escape SM neurons were found in the pretectum, and only seven looming sensory neurons were found in the thalamus, across all fish.) We found that the fraction of SM neurons that were significantly correlated with sensory neurons within the same area was substantially lower for the thalamus and pretectum than for the tectum (all less than 8%, *SI Appendix*, Fig. S15*A*), with the exception of pretectum hunting SM neurons, of which 24.62% were significantly correlated with pretectal prey sensory neurons. This suggests that the pretectal hunting SM neurons are integrating input from the nearby prey sensory neurons, which raises the question of why two different sensorimotor integration sites would exist for prey stimuli. We hypothesized that a different kind of sensory input might be extracted in the pretectum and conveyed to the tectal hunting SM neurons. We found that indeed, the majority of hunting SM neurons in the pretectum had significant partial correlations with tectal hunting SM neurons, (*SI Appendix*, Fig. S15*B*), suggesting that hunting SM neurons in these two areas are connected.

### Tectal Sensorimotor Neurons Respond Specifically During One Behavior.

**Fig. 6.**
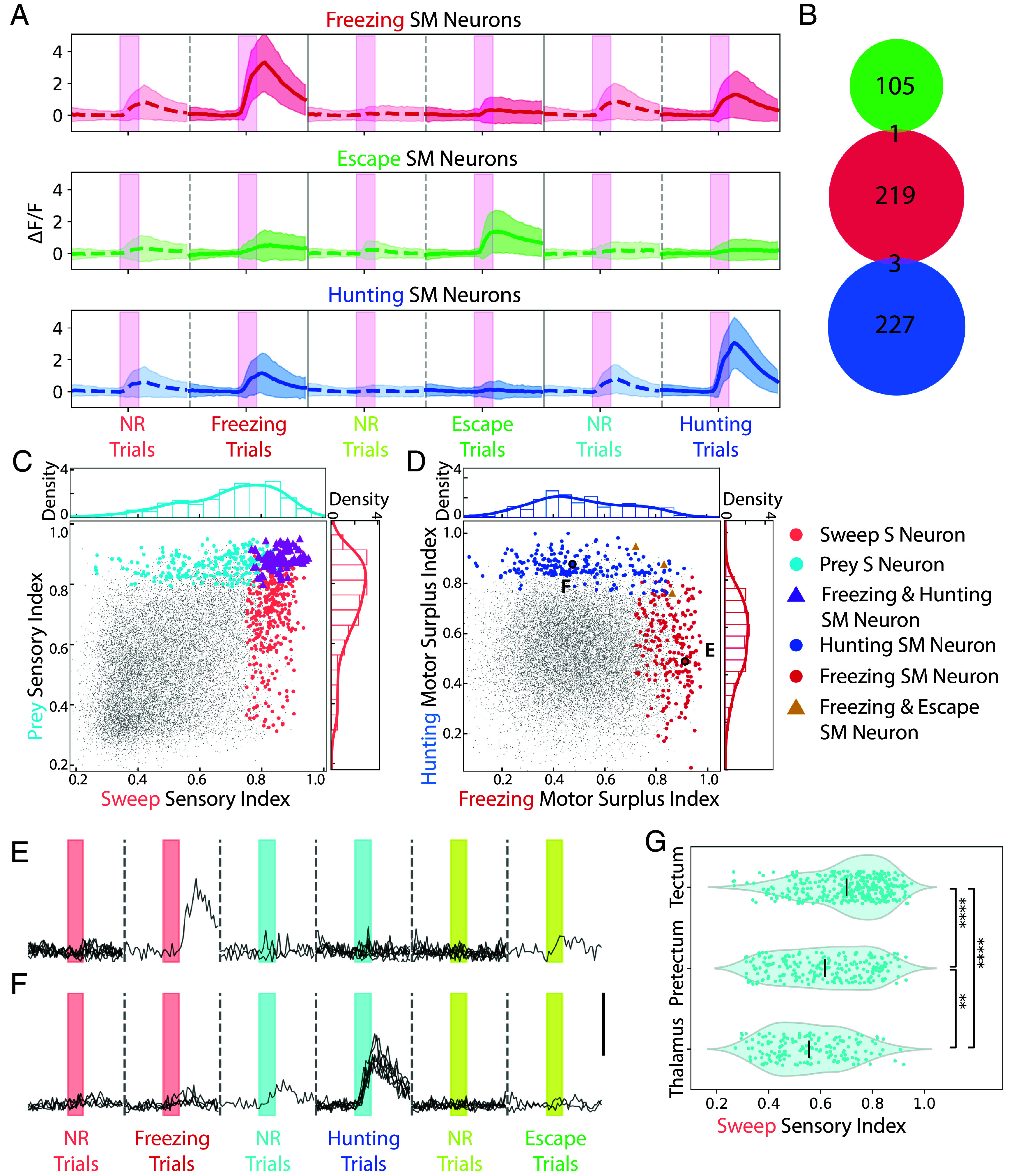
Functional segregation of the different types of sensorimotor neurons in the tectum. (*A*) Activities of tectal SM neurons during motor response or no response trials. Pink bars represent 4 s stimulus presentation. n = 7 larvae. Shading represents SD. (*B*) Venn diagram of the three SM neuron populations in the tectum. Green = escape SM, red = freezing SM, and blue = hunting SM neurons. (*C*) The distribution of the tectal sweep (light red) and prey (light blue) sensory neurons on the sweep and prey sensory index. Purple triangles: neurons belonging to both populations. (*D*) The distribution of tectal freezing (dark red) and hunting SM neurons (dark blue) on the MSI of freezing and hunting. Gold triangle: neuron belonging to both populations. (*E* and *F*) Responses of example tectal freezing and hunting SM neurons from *D*. The scale bar indicates ΔF/F = 3. (*G*) Distribution of the sweep sensory index of prey sensory neurons in the tectum, pretectum, and thalamus. Black lines indicate averages **, *P* < 0.01. ****, *P* < 0.0001, Kruskal–Wallis test, followed by Dunn’s test.

Although the existence of tectal SM neurons for these behaviors has been inferred based on stimulation experiments (27, 48, 49), this dataset gives us the opportunity to assess their functional properties; for example, how do they respond to various visual stimuli and during the different types of behavior? It could be that some of the tectal SM neurons are correlated with multiple behaviors, for example responding in both freezing and escape trials and signal the presence of a predator. To test this, we looked at the responses of each class to the three behaviors and found that on average the populations responded only during their primary behavior (Fig. 6*A*). On an individual neuron level, there was only one tectal neuron that was classified as both an escape + freezing SM neuron and three neurons that were classified as both freezing + hunting SM neurons (Fig. 6*B*).

To look more closely at how the tectum generates separate hunting and freezing sensorimotor populations, we plotted the functional distributions of the prey and sweep sensory neurons in the tectum and saw that these populations were highly overlapping (Fig. 6*C*). In contrast, SM neurons for hunting and freezing had divergent responses (Fig. 6*D*–*F*). How is the tectum able to go from overlapping sensory to divergent sensorimotor populations? In the case of the prey stimulus, other areas such as the pretectum and thalamus also receive input from prey-selective retinal ganglion cells (15) and contain prey sensory neurons (Fig. 2*C*). We plotted the sweep SI value of prey sensory neurons in the pretectum and thalamus, found that these neurons are less responsive to the sweep stimulus (Fig. 6*G*), and may be more selective for prey than the prey sensory neurons of the tectum. We observed a similar divergence for escape vs. freezing sensorimotor populations (*SI Appendix*, Fig. S16). These results suggest that the hunting, freezing, and escape sensorimotor pathways have already diverged at the level of the tectum.

### Downstream Areas Contain Sensorimotor Neurons for the Three Behaviors.

To investigate the flow of visuomotor information from the tectum to other areas, we next looked at the anatomical distribution of SM neurons within three areas likely to be downstream of the tectum (29); the NI, thalamus, and pallium. Within the NI, SM neurons were distributed in distinct patterns, with escape SM neurons localized to the posterior isthmi (*SI Appendix*, Figs. S17*A* and S18). We next looked at the functional properties of the NI SM neurons, by plotting each neuron’s MSI. We found that the escape and freezing SM neurons in this area comprised distinct populations, although there was a subset of neurons that were classified as both (10.7% of all NI freezing SM neurons). Varying the SM neuron threshold did not greatly increase the number of freezing + escape neurons (*SI Appendix*, Fig. S19). A smaller population of NI neurons were both freezing and hunting SM neurons (*SI Appendix*, Fig. S17*C*).

Like the NI, the thalamus also contains all three types of SM neurons, but the freezing and escape populations are restricted to medial thalamus, suggesting this could be a predator-specific region (*SI Appendix*, Fig. S17 *D–F*). In contrast, within the dorsomedial pallium, the homologue of the mammalian amygdala (52), we identified only freezing and escape and no hunting SM neurons (*SI Appendix*, Fig. S17 *G*–*I*). Our analysis of these three areas suggests that after segregation of the pathways in the tectum, the freezing, escape, and hunting pathways continue to be segregated, although there are a few neurons in the NI and thalamus that are part of two pathways.

## Discussion

Here, we developed an imaging-compatible behavioral assay for the innate, visually evoked freezing response in larval zebrafish, and used this assay as well as the established hunting and escape behaviors to map the neural circuits for detection of visual stimuli and selection of visual behaviors. Not surprisingly, the tectum contained large populations of sensory neurons for all three visual stimuli, and these neurons are likely integrating tectal retinal ganglion cell inputs to respond to moving objects in the visual field (*SI Appendix*, Fig. S20). The sensory neurons may then provide input to tectal SM neurons of the corresponding behavior, which could also receive other inputs that reflect fear, satiety, locomotor, or other internal states (24, 25, 53).

Our findings that the tectum contained both sensory and SM neurons for the three stimuli/behaviors and that the SM neurons there were highly correlated with behavior suggest that the tectal SM neurons are receiving sensory information. Our partial correlation analysis showed that freezing and hunting SM neurons in the tectum have significant correlations with the corresponding tectal sensory neuron populations. Therefore, it is possible that these SM neurons receive sensory neuron input, perhaps using it in conjunction with the tectum’s map of visual space (26) to determine the location of the object. Interestingly, we do not find the same evidence of within-tectum sensory correlations for escape SM neurons (Fig. 5*J*). It may be that escape is different from the other two behaviors, in that when an animal is hunting an object or freezing in order to evaluate it, knowledge of its position in the visual field is crucial, whereas during escape, the precise predator location may not be as important as executing an evasive movement roughly away from the threat.

Although tectal freezing and hunting SM neurons seem to be receiving sensory input and are correlated with behavior, we cannot conclude that they are making the decision to freeze or hunt, as they may also receive input from other areas that actually make the decision. In addition, the tectum may not be the only place where SM neurons receive sensory input. We also found that pretectal hunting SM neurons are significantly correlated with the pretectal prey sensory population (*SI Appendix*, Fig. S15*A*). Pretectal prey sensory neurons are more specific for prey stimuli than their tectal counterparts (Fig. 6*G*), so the pretectal hunting SM neurons may be using the information from the prey-selective retinal arborization field 7 (15) to identify prey and initiate eye convergence. Pretectal SM neurons could then activate tectal hunting SM neurons, which would integrate the information on object location from the tectal sensory neurons to precisely target prey. Indeed, hunting command-like neurons have been identified in the pretectum, and they have projections to the anterior optic tectum (32). It is likely that these neurons are part of the pretectal hunting SM population, and we indeed see that a large percentage of pretectal hunting SM neurons are correlated with the tectal SM population (*SI Appendix*, Fig. S15*B*). These data support a model where identification of prey occurs in the pretectum, and this information is transmitted to tectal hunting SM neurons, which encode precise prey-directed swims based on object location.

It is also entirely possible that tectal freezing SM neurons receive input from other areas such as the thalamus, which also contains freezing SM neurons, and that the decision to freeze is actually made in the thalamus and then conveyed to SM neurons in the tectum, where object location information can be added. It could also be that tectal SM neurons receive feedback from motor areas in the hindbrain, and it is these other areas could actually decide whether the animal is going to behave. Furthermore, it remains unclear how SM neurons read out sensory neuron activity. It is possible that the neuronal dynamics over time or average activity of the entire population of sensory neurons is important, as our current analyses cannot elucidate or distinguish between these potential mechanisms. An additional caveat is that we cannot infer direct monosynaptic connectivity from the analysis of correlations. Further studies will be required to identify the direction of information flow and the functional roles of each area in detecting stimuli and generating visual behaviors.

In addition to the tectum, we also found significant populations of hunting, freezing, and escape SM neurons in the thalamus and NI (homologue of the parabigeminal nucleus), while the dorsomedial pallium (homologue of the amygdala) contained only freezing and escape SM neurons. In terms of escape, our results agree with recent studies in zebrafish that found looming stimuli evoke responses in the tectum, thalamus, and pallium (45, 54), although escape behavior was not recorded in these studies, so escape SM neurons could not be identified. Our findings are also consistent with studies in mice where optogenetic stimulation of the thalamus, parabigeminal nucleus, and amygdala evoked freezing and escape (33, 55), suggesting that the neural circuits for these important innate behaviors are deeply conserved. In mice, the LP region of the thalamus has been implicated in freezing and the parabigeminal nucleus in escape, based on the fact that optogenetic activation of these areas biases animals toward these behaviors (33). In contrast, our data show that these areas contain a significant number of SM neurons for both defensive behaviors. It may be that, rather than mediating a single behavior, these areas are involved in functions relevant to multiple behaviors, such as stimulus selection (56), or object identification (30). The exact role of these areas in these behaviors, as well as the locus of behavioral decision-making, remains to be determined.

## Methods

Larvae were presented with prey, sweep, and looming stimuli while heart, eye angles, and tail movements were recorded. Hunting, freezing, and escape trials were defined based on eye convergence, heart rate decrease, or fast tail movement during the stimulus period, respectively. 2-photon imaging data were collected from 14 planes at 2 Hz per volume, and cell bodies were segmented. Sensory and Motor Surplus Indices were calculated for active neurons. For detailed Methods, see *SI Appendix*.

## Supplementary Material

Appendix 01 (PDF)

Movie S1. **Sweep stimulus suppresses spontaneous swims**. Swimming was recorded from above at 200 frames per second. The stimulus was a 15° diameter dark disk moving at 60°/second on a red background.

Movie S2. **Sweep stimulus causes a reduction in heart rate**. The stimulus was a 15° diameter dark disk moving at 60°/second on a red background. Heart rate was recorded from the side at 100 frames per second.

Movie S3. **Prey stimulus triggers hunting behavior**. Eye and tail movements recorded from above at 200 frames per second, in response to a 4° diameter UV dot moving at 120°/second for 6 seconds. For imaging experiments, the speed was 60°/second and duration was 4 seconds.

Movie S4. **Looming stimulus triggers escape behavior**. Tail movements were recorded from above at 200 frames per second, in response to a dark disk expanding to 60° in diameter.

## Data Availability

Code has been deposited in GitHub (https://github.com/SemmelhackLab/Freezing_Code/tree/main) (57). Behavioral videos, visual stimulus information, segmented neuron information, and neuronal delta F/F traces are available on figshare (58). All other data are included in the manuscript and/or supporting information.
