# Supplementary material for: The visuomotor transformations underlying target-directed behavior
Source: Proc Natl Acad Sci U S A. 2025 Mar 24;122(13):e2416215122. doi: 10.1073/pnas.2416215122 (PMC12002292; doi:10.1073/pnas.2416215122)
Supplement: Supplementary file 1 — Appendix 01 (PDF) [file pnas.2416215122.sapp.pdf]

Methods

Code Availability

Key code, including neuronal data preprocessing, SI and MSI calculation, sensory and sensory motor neuron selection, and behavioral analysis pipeline (heart rate analysis, bradycardia detection, hunting detection, tail bout detection, and escape classification) is available on github: https://github.com/SemmelhackLab/Freezing_Code/tree/main

Animals

Zebrafish larvae were raised in Danieau's solution in a petri dish inside an incubator set to a standard 14:10 hour light cycle at 28.5°C. All the larvae used in this study carried homozygous *mitfa* skin-pigmentation mutation (nacre). Tg(elavl3:H2B-GCaMP6s) larvae were used for the imaging experiments. All animal experiments were conducted with approval from the Animal Ethics Committee of Hong Kong University of Science and Technology.

Behavioral Experiments

Each larva was embedded at 5 or 6 days post fertilization in 2% low melt agarose on a Rinzl plastic coverslip (Delta Microscopies). Larvae that were embedded on day 6 were fed 1 mL of a dense paramecium culture per petri dish on day 5. After solidification, the agarose around the head and tail was then removed to free the eyes and the caudal half of the tail. The embedded larvae were allowed to accommodate overnight before the experiment.

Behavioral experiments were conducted in a chamber where stimuli were projected on one wall and high-speed cameras recorded from above and below, as previously described[1,2]. Before the experiment, the coverslip with the larva was inserted into a 3D printed resin scaffold connected to an XYZ manipulation stage (Heng Yang Optics). The larva was then secured inside an arena filled with Danieau's solution. The arena is a rectangular tank made of transparent acrylic board, with the frontal wall made of Teflon fabric serving as a screen. The dimensions of the arena were 60×80×30 mm. The distance between the screen and the fish was 10 mm. The top camera (Photon Focus, MV1-D1312E-160-CL-12) recorded the eye and tail movement of the fish, while the left camera (HIK Robot MV-CA013-21UM/UC 130) captured the heart rate. The frame rate was 200 frames per second for the top camera and 100 frames per second for the left camera. Recording from the two cameras and the stimulus display were synchronized with custom Python scripts. For both the behavioral and imaging experiments, a customized LightCrafter (DLP® Light Crafter™ 4500 modified by EKB Technologies Ltd was used for stimulus projection, placed

13 cm away from the frontal screen. The wavelengths of the LEDs were 385, 415, 470, 590 nm for UV, blue, green, and red, respectively. The light intensity of the stimulus or the colored background was 886 nW/cm^2, consistent across colors.

Before the start of stimulus presentation, the fish were allowed to accommodate for 5 minutes. Stimuli were presented in pseudo-random order. After each stimulus display, there was a trial with no stimulus, and the inter-trial interval was 1 minute. The duration of each trial was 22 seconds, and the stimulus was shown during the 9th -13th second, unless specified otherwise.

Visual Stimuli

Visual Stimuli were designed in Python using PsychoPy[37]. For the behavioral experiments to optimize the sweep stimulus (figures 1 and 2), the stimulus was a dark disk moving horizontally back and forth across the red background (-60° to 60° in azimuth). The starting azimuth of the stimulus was either -60° or 60° alternating among trials to avoid habituation. The elevation of the stimuli was 5° above the midline. The diameter of the stimulus was 15°, and the speed was 60° per second, unless otherwise stated.

For the imaging experiments, the sweep stimulus was a dark disk with a diameter of 15° and a speed 60°/s, on a red background. The prey stimulus was a 4° UV dot with the same horizontal movement trajectory as the sweeping stimulus, but an elevation of 15° to promote hunting behavior. The looming stimulus was a dark expanding disk on the same red background, starting from 6° and ending in 60° in diameter. The expansion size-to-speed ratio (l/v) was 60ms. After reaching the maximum size, the looming stimulus remained stationary until the 4 second stimulus period was over. The looming disk was presented in the center of the visual field (azimuth = 0°) and the elevation of the center of the disk was -20° (Supplementary figure 2A), to promote escape responses. All trials lasted for 22 seconds in total. We presented eight sweep stimuli, followed by eight prey stimuli, followed by eight looming stimuli, with an interstimulus interval of 2 minutes.

Heart Rate Analysis

To analyze the heart rate, an ROI of around 100x100 pixels that contained the heart was manually cropped, and rhythmic pixels within that ROI were selected (figure 1B, left and middle). The first criterion for rhythmic pixels was having stable local maximum and minimum intensity values (each local max>(.75* last local min + .25*last local max), and each local min<(.75*last local max +.25*last local min)), and the second was having a stable heart rate over the whole trial (standard deviation of less than 0.55). If the number of rhythmic pixels was less than 5%, the trial would be excluded from further analysis. The intensity trace of each pixel was gaussian filtered (figure 1B, right), and the heart rate for that

pixel was calculated as the reciprocal of the inter-beat interval. Heart rates from all the rhythmic pixels were averaged to calculate the heart rate for the trial. Heart rate was normalized by dividing by average heart rate in the 6 seconds before stimulus onset.

Bradycardia Identification

To identify bradycardia, a dynamic ceiling, or local presumptive max heart rate, was calculated[3]. The ceiling was acquired by applying a maximum filter with a 300-frame window to the median filtered heart rate trace. The distance between the original heart rate and the ceiling, termed heart-rate-to-ceiling, was calculated for every frame to examine the local decrease in heart rate. If the heart-rate-to-ceiling of the frame was larger than 3 standard deviations of the heart-rate-to-ceiling of all the frames from no stimulus trials, the frame was considered a bradycardia frame. When the bradycardia persisted for 50 frames (500 ms), the period was annotated as a bradycardia episode.

Eye and Tail Tracking

Eye and tail tracking were conducted by Ztrack in Python. In brief, the contours of both eyes were found by multi-threshold binarization. The binocular eye angle was calculated from the image moment of the contours. To detect eye convergence, the distribution of binocular eye angles was fitted with a kernel density estimation and the location of the first local maximum within 20-50° was chosen as the threshold for eye convergence episodes of the corresponding fish. If no local minimum was found, trials were checked manually for convergence.

For tail tracking, a skeleton of tail with 20 nodes was tracked from the binarized image. The tail curvature was determined as the average of the nodal angle of the last 10 nodes. To detect swimming bouts, hysteresis thresholding was applied to the derivative of the tail curvature with respect to time. Bouts near each other would be merged as one bout and bouts that were less than 8 frames were discarded. The swimming probability of each frame was the probability that a swimming bout was detected at that point across all trials.

Escape Classification

To identify escape bouts, all swimming bouts detected in the dataset were processed by Principal Component Analysis (PCA), followed by K-Means Clustering. 8 features of each swimming bout were dimensionally reduced by PCA: the curvature, angle of the tail tip point, angle of the tail middle point, mean and max velocity of tail movement, frequency of tail movement, duration of the bout, and the integral of the tail bouts. The first 3 PCs explained over 90% of the variance. K-means clustering was applied to the first 3 PCs of all tail bouts and 5 clusters were formed. The cluster with high frequency, high velocity, and short bout duration was selected as escape bout cluster. To examine the accuracy, 80 new bouts were manually annotated, and the classification showed 92% accuracy compared to manual

annotation.

Behavioral Categorization

The behavior of fish in each trial was classified into one of 5 types: Freezing, Hunting, Escape, Spontaneous Swim, and No Response (figure S3B). To be classified as one type of behavioral response, phenotypical signatures of the behavior had to be detected, and the behavior had to start during the 4 seconds after stimulus onset. The classification criteria are shown in Supplementary figure 3A. To be classified as a freezing trial, there must be a bradycardia episode (heart-rate-to-ceiling larger than 3 SD for 500 ms, Supplementary figure 3A, upper panel) starting during the stimulus period, and no eye or tail movement. For escape, an escape bout (high frequency and velocity, within the escape cluster, Supplementary figure 3A, middle panel) starting during the stimulation period was required. For hunting, eye convergence above the first local minimum of eye angle for the animal (Supplementary figure 3A, lower panel) starting during the stimulus period was required. For spontaneous swim, there had to be a non-escape bout with no escape bout or eye convergence during the stimulation. Finally, trials with none of the above types were considered no response trials. Trials with a mixture of behaviors (e.g. freezing followed by escape) during the stimulus period were excluded in further analysis.

Calcium Imaging Acquisition

2-photon calcium imaging was conducted with a Nikon upright 2-Photon microscope (Nikon A1 MP multiphoton) with a custom-built behavior setup. To achieve multi-plane imaging, an Electrically Tunable Lens (Optotune) was added between the microscope and the 25x Nikon Objective. The ETL was synchronized with the imaging system and triggered every frame. The wavelength of the laser was set to 920 nm. Images of 512 x 512 pixels were recorded at 2 volumes/second from 14 planes, with around 15 microns between planes.

The imaging chamber was similar to that used in the behavioral experiments. The fish, embedded on plastic cover slip and secured to the XYZ stage, was placed 1.5 mm beneath the objective front lens. Both the front lens and the fish were immersed under water held in a rectangular arena made of transparent acrylic board, except the front wall made of Teflon fabric and serving as the screen. The distance between the screen and the fish was 10 mm, and the screen area was 70° high x 140° wide, with the larva positioned at 0°. A LightCrafter (DLP Lightcrafter 4500 MKII) was placed in front of the screen, 15 cm away, to project the virtual stimulus. Two sets of cameras were placed around the arena. A bottom-view camera (Photonfocus, MV1-D1312E-160-CL-12) and light record the eyes and tail at 200 frames per second. A side camera (HIK Robotics, MV-CA013-21UM) recorded the heart rate of the fish at 100 frames per second.

The presentation of the stimulus, the recording of cameras, and the tuning of the ETL were all synchronized with the calcium imaging session by Labjack (Labjack U3), custom-built circuits, and custom Python scripts.

Calcium Imaging Analysis

All calcium imaging analyses were performed with custom-written Python code. Motion correction and neuron segmentation were conducted with suite2p[4]. Volumetric imaging data from three fish was used to train a suite2p classifier to segment ROIs. For each plane, around 800-3000 ROIs were segmented, and ROIs larger than 300 μm^2 or smaller than 30 μm^2 were discarded. ΔF/F of each neuron were calculated as $(F(t)-F_0)/F_0$ in each trial, where F_0 was calculated as the average intensity over the 4 seconds before stimulus onset, and F(t) was the intensity trace of each neuron.

All calcium imaging registration was done with Advanced Normalization Tools (ANTs). Software and all reference volumes were downloaded from mapZebrain[5]. The registration was performed in 3 steps: 1) 3d registration from the z-stack to the atlas. 2) 2d registration from the calcium imaging video to the z-stack. The average calcium image was used as the moving image and the reference image was the best-matched plane chosen from the z-stack by template matching (OpenCV). 3) Transformations were applied to the coordinates of segmented neurons. Anatomical regions of each registered neuron were identified by the masks downloaded from mapZebrain.

Selection of Active Neurons

Neurons with either sensory- or motor-related activity were selected from all the segmented cell bodies using linear regression. Sensory-relevant activity was represented by a *sensory regressors* (Regressor_S , one for each stimulus) built by a box car function over the stimulus presentation window (9-13s), then convolved with the GCaMP6s kernel (i.e., an exponential decay $e^{-\frac{t}{\tau}}, t > 0$ with a time constant τ = 7s[6]). The motor-related activity was similarly represented by *visuomotor regressors,* Regressor_{SM} . To construct a visuomotor regressor, we build a 1 second box car function starting from the behavior onset (i.e., bradycardia without movement, escape tail bouts, and eye convergence movements as the onsets of freezing events, escape events and hunting events, respectively). We then shifted the box car by -0.5 seconds and convolved it with the GCaMP6s kernel[6]. Only motor events that started within the stimulus presentation window (9-13s) were used to construct the visuomotor regressor. For each neuron, we regress its activity in each trial to one of the sensory and motor regressors. Neurons with at least 30% of trials having a slope>0.35 and R^2 >0.36 to any sensory or visuomotor regressor were considered active neurons. In the end, we found 34% of segmented cell bodies were classified as active neurons and used this set for

subsequent analysis.

Sensory Index Calculation and Sensory Neuron Identification

We used a similar method to identify sensory neurons as in Chen *et. al.*[7] In brief, if the information encoded in a neuron is purely sensory, its response to the repeatedly presented stimulus across trials should be similar and thus having a highly periodic activity. Therefore, we can select a sensory neuron using periodicity as a sensory index, which measures how similar the neuron's responses are across trials.

To identify sensory neurons for a given stimulus, we first selected neurons that responded to the stimulus within the population of active neurons, using the same $\mathrm{Regressor}_S$, and took the top 10% most correlated neurons (Pearson's correlation coefficient). To calculate the periodicity, we form a Trace_{avg} by tiling or concatenating a neuron's trial-average response to the stimulus over each of the trials (figure 2B). Then the periodicity, i.e., the sensory index (SI), was calculated as the square root of the variance ratio between Trace_{avg} and the original activity Trace (see equations below). The periodicity of each neuron was calculated per stimulus; thus, each neuron had 3 sensory indices.

$$SI = \sqrt{\frac{var(Trace_{avg})}{var(Trace)}}$$

$$var(Trace) = \frac{\Sigma_{t=1}^{T}\left(Trace(t) - \overline{Trace}\right)^2}{T}, \quad \overline{Trace} = \frac{1}{T}\sum_{t=1}^{T} Trace(t),$$

$$where\ Trace(t)\ (t = 1,2,\ldots,T)\ is\ the\ trace\ value\ at\ frame\ t.$$

We define the top 15% of neurons by sensory index as sensory neurons. The selection was conducted for each stimulus, and one neuron could be selected as a sensory neuron for more than one stimulus. Neurons that were not spatially colocalized across fish were eliminated by a *Spatial colocalization test,* similar to Spatial p-value filtering[8]. Briefly, for a given neuron *i,* we compute the average distance of this neuron to the nearest neuron (other than itself) within the same category in each fish. This average distance is then compared with the null distance. The null distance is the distance of neuron *i* to a neuron randomly sampled from all the active neurons combined across all fish (recall that the fish is registered to a standard brain atlas). The random sampling of neuron and the null distance were repeated 5000 times to build a null distribution, which is then fitted to a normal distribution. The neuron *i* is considered anatomically colocalized across fish if the average nearest distance falls under the 2.5% normal distribution percentile (i.e. p-value < 0.025).

Motor Surplus Index Calculation and Sensorimotor Neuron Identification

The calculation of Motor Surplus Index was inspired by the motor decomposition method in Chen et al.[7]. Firstly, for each active neuron, a trace surplus was calculated by subtracting the average trace from the original trace (figure 3C). Then, the Pearson's correlation coefficient between the trace surplus and the visuomotor regressor ($\mathrm{Regressor}_{SM}$,

same as in "**Selection of Active Neurons**" but concatenated across trials) was calculated. The correlations are linearly scaled to a motor surplus index (MSI) between 0 and 1, using the min and max correlation in each fish. Note that this scaling is only for better visualizing results across fish, but has no effect on sensorimotor neuron identification. The MSI of each neuron was calculated per behavioral response (freezing, escape, and hunting) using recordings during the corresponding stimulus window (sweep, looming, and prey), resulting in 3 MSIs for each neuron. For each behavioral response and each fish, the top 3% of the neurons with the highest MSI are selected as the preliminary SM neurons. The Spatial colocalization test (as described in "**Sensory Index Calculation and Sensory Neuron Identification**") was applied to further select neurons that are anatomically colocalized across fish (i.e., with a p-value < 0.025) and were used as the final SM neurons for further analysis. Out of all the preliminary SM neurons for each behavior, 74.5%, 74.3% and 74.3% of them passed the Spatial colocalization test.

Behavior Prediction using Freezing SM Neurons

The behavior prediction using freezing SM neurons was conducted on a leave-one-out basis. We held out one trial and used the remaining trials to select SM neurons based on the top 3% MSI criteria. The behavior prediction is determined by comparing the activity of selected SM neurons in the held-out trial with the average activity in behavioral trials and in non-behavioral trials. In particular, we use the maximum of the average activity trace in a trial as the feature. If this maximum in the held-out trial is closer to the maximum value of the behavioral-trial average, we will predict the held-out as a behaviorial trial, and vice versa. Under this method, the held-out prediction could only be conducted when there are at least one behavioral and one non-behavioral trials after held-out. Therefore our results are from a subset of held-outs for certain fish. For example, for a total of 8 sweep trials in one fish, if there was only one behavioral trial, then there would be 7 held-outs that we can make the prediction.

The prediction accuracy for each possible held-out trial (100% or 0%, left column, Supplementary figure 9D and 10) is compared per held-out trial with the accuracy using *null SM neurons.* Null SM neurons were selected similarly, using sensorimotor regressors built according to trial-permuted behavior labels, for the activity in the seven remaining trials. Since there are multiple ways to permute the behavior labels, a different set of null SM neurons will be selected for each permutation. For example, if there were 8 trials in total and 2 trials are behavioral trials, if the held-out trial is a behavioral trial, there would be 7 behavior label permutations and sets of null SM neurons. If the held-out trial is a non-behavioral trial, there would be 21 permutations.

For each permutation and corresponding set of null SM neurons, we use the same method of trace maximum comparison as for the real SM neurons to make the behavior

prediction for the held-out trial. We then average this accuracy across permutations to get the null prediction accuracy (right column, Supplementary figure 9D and 10).

Single-photon optogenetic stimulation

Zebrafish larvae were embedded with eye and tail were freed, and placed in the behavioral chamber. An optical fiber, 50 µm (M14L01 Thorlabs) or 105 µm in diameter (M15L01 Thorlabs), was held about 0.5mm above the head and guided the light from a blue light fiber-coupled LED (M470F4, Thorlabs). We used 0.06 mW or 0.26 mW light power, measured at the fiber tip. The position of the optic fiber was controlled using an XYZ manipulation stage (Heng Yang Optics). We targeted the anterior and posterior tectum and stimulated each spot with 10 trials. Each trial contained a 5 s stimulation period and a 5 s interval between each trial. Behavior was recorded at 200 fps and manually annotated for eye convergence and escape tail movements.

Partial Correlation Calculation between Behavior Residuals of Sensory and Sensorimotor Neurons

In each fish, we used response during the presentation of a given stimulus concatenated across trials to represent the activity of each sensory neuron or SM neuron. For example, if there are 8 sweeping stimulus trials and 8 frames during each stimulus presentation. S is a $[64 \times n]$ matrix and SM is a $[64 \times m]$ matrix, and *n* and *m* are the numbers of sensory and SM neurons, respectively. Neurons that are categorized as both S and SM are excluded from this analysis (between 0-16.7%, or 1-3 neurons across fish) to avoid issues due to self-correlated pairs in later analysis.

For the concatenated response of a sensory neuron S_i (i=1,2,...,n) and a SM neuron SM_j (j=1,2,...,m), i.e., a column in the above S or SM matrices, the behavior average and non-behavior average are calculated by averaging the responses of behavioral and no-behavioral trials, respectively (Supplementary figure 12A). $S_{i,res}$ and $SM_{j,res}$was calculated by subtracting the behavior average and non-behavior average for each trial, according to its behavior type, from the original responses S_i and SM_j (Supplementary figure 12A). For a pair of S neuron *i* and SM neuron *j*, the partial correlation $\rho(S_i, SM_j | B)$ is the Pearson's correlation coefficient between $S_{i,res}$ and $SM_{j\,res}$[9].

Testing Significant Partial Correlations between Sensory and Sensorimotor Neurons Given Behavior

For each SM neuron *j* in optic tectum (or nucleus isthmi, NI), we quantified its correlation strength with tectal sensory neurons by the 90th percentile of the partial correlations $\rho(S_i, SM_j | B)$ (Supplementary figure 12C, right). To obtain a null distribution of these partial correlations, we permute the $SM_{j,res}$ trace across trials, while keeping the 8-frame temporal

structure within each trial (Supplementary figure 12B). There are thus in total $k!$ possible permutations, where k is the number of trials. After each permutation, we similarly compute the partial correlations between the permuted $SM_{j,res}$ and all the tectal sensory neurons, and calculate a 90th percentile (Supplementary figure 12C, left). Collecting such partial correlation percentiles across the $k!$ permutations gives a null distribution for the SM neuron's correlation strength (Supplementary figure 12D) and an empirical p-value can be computed as $x/k!$, where x is the number of permutations resulting in a partial correlation 90th percentile that is equal or larger than to the percentile for the original $SM_{j,res}$. The p-values of SM neurons (in the same brain region) from all fish are aggregated and adjusted together for multiplicity into q-values to control the False Discovery Rate (FDR) using the Benjamini-Hochberg procedure. We quantified the percentage of SM neurons with significant q-values (<0.05) in figure 5k. Percentage-by-chance is calculated as a control by using the trial-permuted SM_{res} in place of the real ones in the above analysis steps. We collect such percentage-by-chance values over 1000 permutations. The 95th percentile of these 1000 percentage-by-chance values is 0 for all three types of SM neurons from both optic tectum (OT) and nucleus isthmi (NI).

To evaluate the potential effect due to shared optical noise, we calculated the percentage of tectal SM neurons with significant partial correlation to a surrogate tectal sensory population (S' neurons) in Supplementary figure 14. To obtain the S' neurons, we replace each tectal S neuron with the nearest neuron in the same imaging plane with an SI +/- 0.05 from the average SI of tectal neurons in that fish. Average SI for tectal neurons ranged from 0.39-0.58 in the seven fish. The average distance between each S neuron and its S' surrogate for sweep, looming, and prey stimulus are 6.2 (± 2.7), 11.0 (± 9.1), 7.5 (± 4.9) µm (Mean ± SD), respectively. In Supplementary figure 14B, we selected surrogate pretectal SM' neurons in the same imaging plane and with an MSI +/-0.05 from the average value for pretectal neurons in that fish. Average hunting MSI was in the range of 0.53 - 0.60 for pretectal neurons in the seven fish.

Statistics

Figure 1D, S1B, C: Mann-Whitney U test.

Figure 1G, Figure 6G, S1E, G, H, I, S19: The Kruskal-Wallis test, followed by Dunn's test (Bonferroni correction)

Figure 5J, S9, S10, S11, S13, S14, S15: One sided Wilcoxon signed-rank test.

P-value <= 0.0001 was denoted by "****"

P-value <= 0.001 was denoted by "***"

P-value <= 0.01 was denoted by “**”

P-value <= 0.05 was denoted by “*”

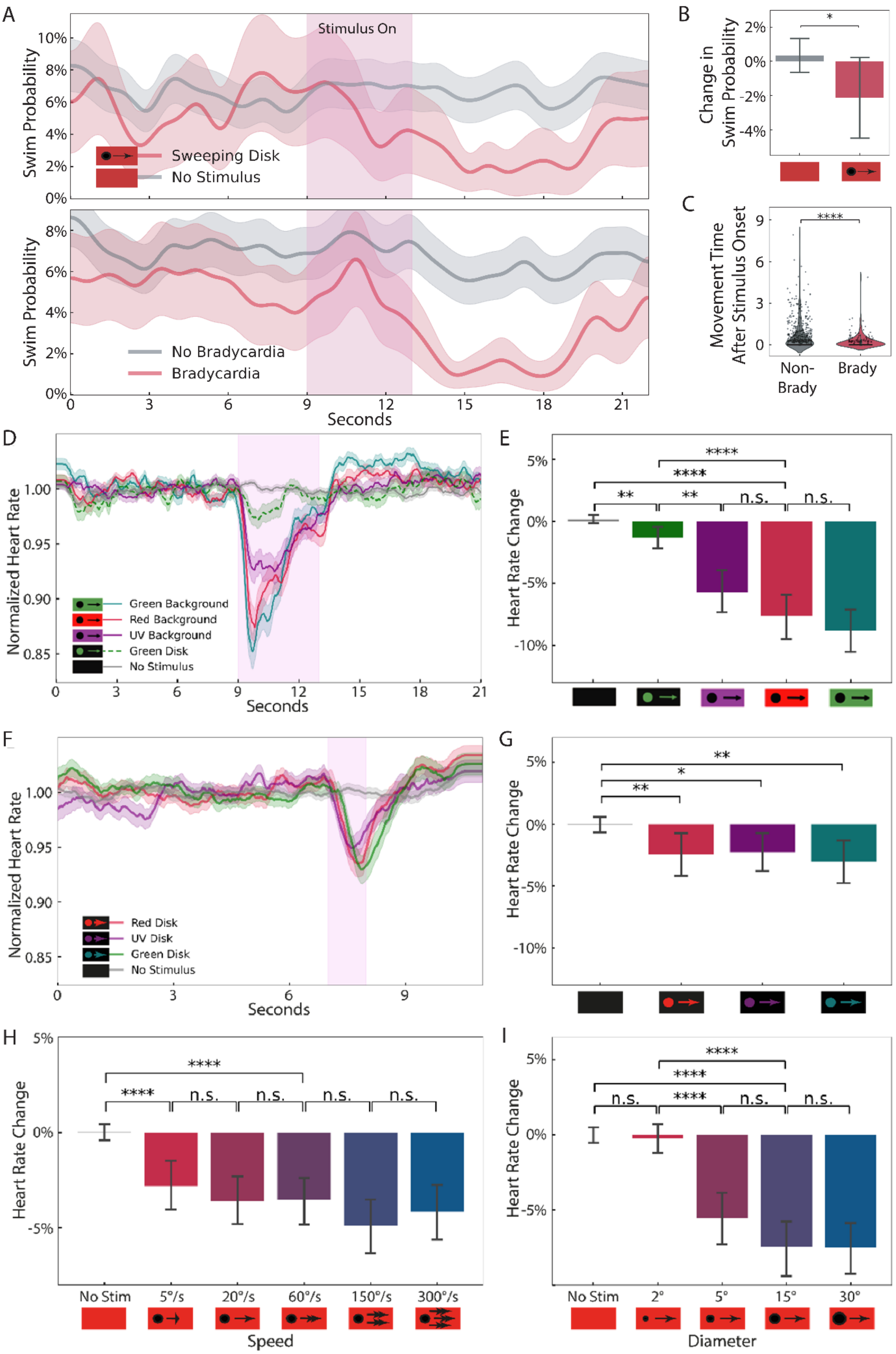

A
Stimulus On
Swim Probability
Sweeping Disk
No Stimulus
No Bradycardia
Bradycardia
Seconds
B
Change in Swim Probability
C
Movement Time After Stimulus Onset
Non-Brady
Brady
D
Normalized Heart Rate
Green Background
Red Background
UV Background
Green Disk
No Stimulus
Seconds
E
Heart Rate Change
n.s.
F
Red Disk
UV Disk
Green Disk
No Stimulus
G
H
No Stim
5°/s
20°/s
60°/s
150°/s
300°/s
Speed
I
2°
5°
15°
30°
Diameter

Supplementary Figure 1: Characterization of the freezing stimulus parameters. (A) Upper: Swim probability over time for sweep (red) vs. no stimulus (grey). Lower: swim probability in trials with bradycardia versus without, for trials with at least one swim bout. Pink bar indicates 4-second stimulus presentation. Shading shows standard error. (B) Change in swim probability after stimulus (9 –22 seconds). (C) Time spent swimming after stimulus onset (9 to 22 seconds). (D) Normalized heart rate over time in response to a sweeping disks of different stimulus and background colors. Pink bar indicates 4-second stimulus presentation (n = 14). (E) Change in heart rate during the 3 seconds after stimulus onset for stimuli in D. (F) Normalized heart rate over time in response to a sweeping disks of different colors on a dark background. Pink bar indicates 1-second stimulus presentation (n = 13). (G) Change in heart rate during the 3 seconds after stimulus onset for stimuli in F. (H) Heart rate change during the 3 seconds after stimulus onset caused by sweeping disks of different speed (n = 13). (I) Heart rate change during the same window for sweeping disks of varying diameters (n = 11). *, $p<0.05$. **, $p<0.01$. ****, $p<0.0001$, Kruskal-Wallis test, followed by Dunn's test. Error bar represents standard deviation.

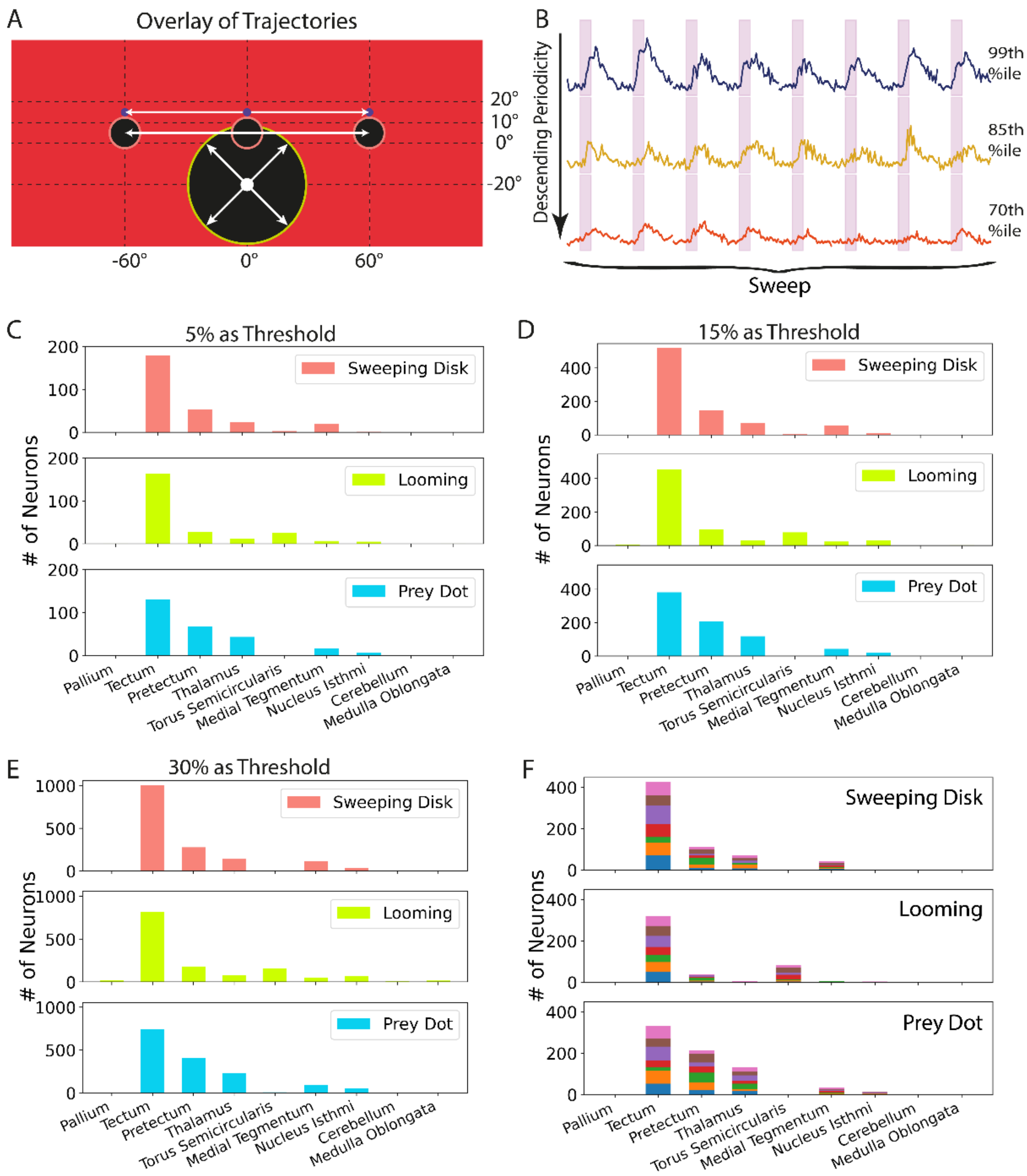

Supplementary Figure 2: Visual stimuli and setting the threshold for sensory neurons. (A) Schematic representing the movement of each stimulus and its relative position on the screen. (B) Example ΔF/F traces during 8 presentations of the sweep stimulus (pink shading) for neurons with sensory indices from the 99th, 85th, and 70th percentile of the sweep sensory index. (C-E) The distrbution of sensory neurons in each brain area based on using the top 10% most correlated neurons and then a threshold of the top 5, 15, or 30% of the neurons by sensory index, as compared to the threshold of 15% used in figure 3. No spatial colocalization filtering was applied for this analysis. (F) The anatomical distribution of each type of sensory neuron across the seven fish, using the top 15% of SI as the threshold. Each color represents the sensory neurons from one fish.

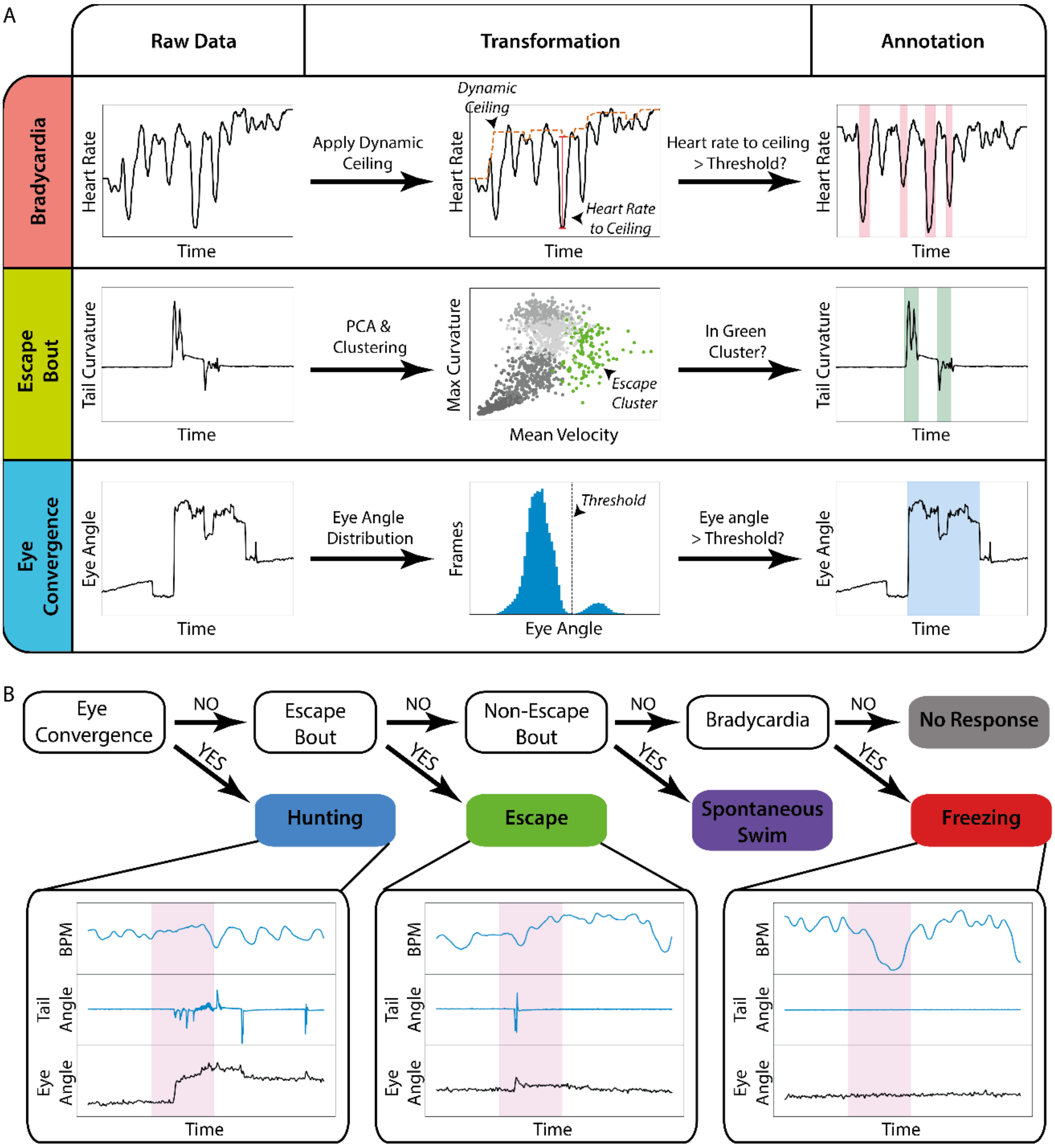

Supplementary Figure 3: The categorization of behavioral responses. (A) Annotation of bradycardia, escape, and eye convergence. Frames were annotated as bradycardia if the heart-rate-to-ceiling distance to ceiling is larger than 3 standard deviations of the heart-rate-to-ceiling distance during no stimulus trials. For escape, any bout belonging to the cluster with high tail velocity and curvature (green) was annotated as an escape bout. To find eye convergence bouts, the distribution of eye angles from each animal was plotted, and the first local minimum larger than the maximum in the distribution was set as the threshold for eye convergence. Any frame with eye angle higher than the threshold was annotated as eye convergence. (B) The sequential pipleline for classification. Examples of each behavior are shown below, with corresponding heart, tail and eye angle data.

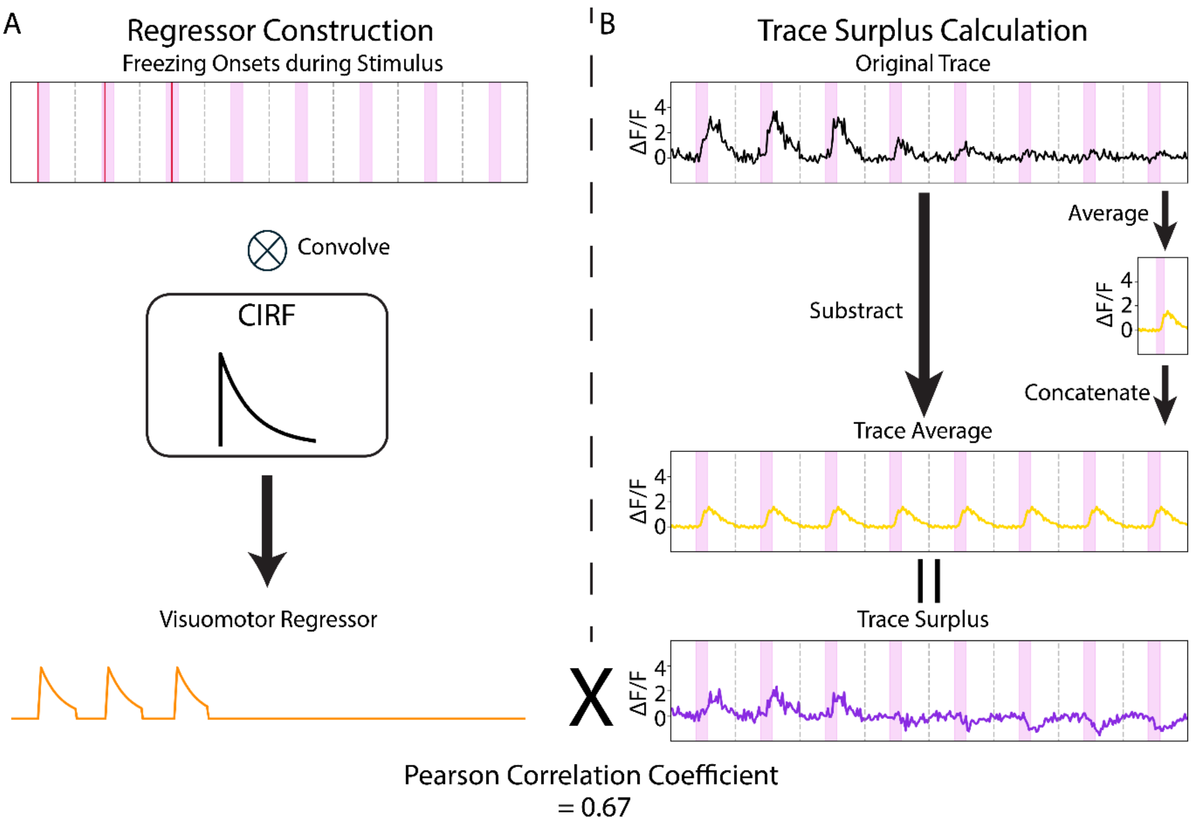

Supplementary Figure 4: Construction of the visuomotor regressors and calculation of correlation score. (A) Example freezing behavior for one fish in the imaging dataset. The freezing regressor was constructed based on the onset of each freezing event (bradycardia + no movement) during the stimulus time period (red lines). The behavior onset time was shifted by -.5 seconds so that the response starts before the behavior, and convolved with a Calcium Impulse Response Function (CIRF) to generate the visuomotor regressor. Regressors for hunting and escape were constructed similarly, using the initiation of eye convergence or the escape bout as behavior onset. (B) Example freezing SM neuron trace from our dataset. The trace average was subtracted from the trace to generate a trace surplus. For this neuron, the Pearson Correlation between the visuomotor regressor and the trace surplus was 0.67.

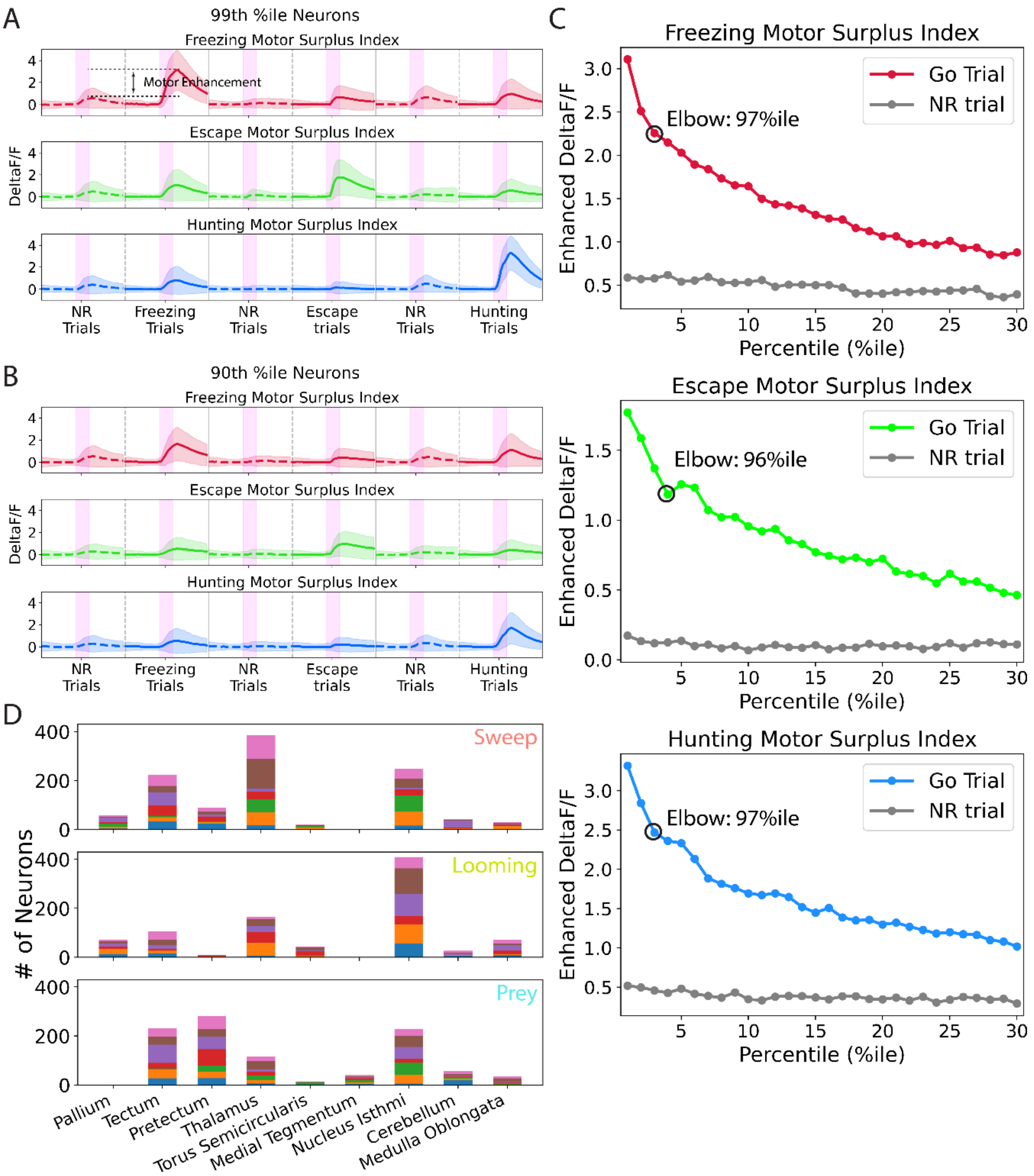

Supplementary Figure 5: Setting the threshold for sensorimotor neurons. (A-B) Average calcium response in behavior and no response trials for sensory-motor neurons from the 99th (A) and 90th (B) percentile of MSI. Motor enhancement is the difference between the peak ΔF/F in response and no response trials. Pink bars represent 4-second presentation of sweep, prey or looming stimuli. n = 7 larvae. (C) Motor enhancement (colored line) for each percentile of neurons on the freezing, escape, and hunting MSI. An elbow was located near the 97th percentile for all three distributions, and this was set as the threshold for SM neurons. Grey line represents peak ΔF/F of that percentile of neurons in NR trials. (D) Anatomical distribution of SM neurons across the seven fish; each color represents SM neurons of that type in one fish. One fish was excluded from the looming SM neuron calculation due to its having no trials without behavior.

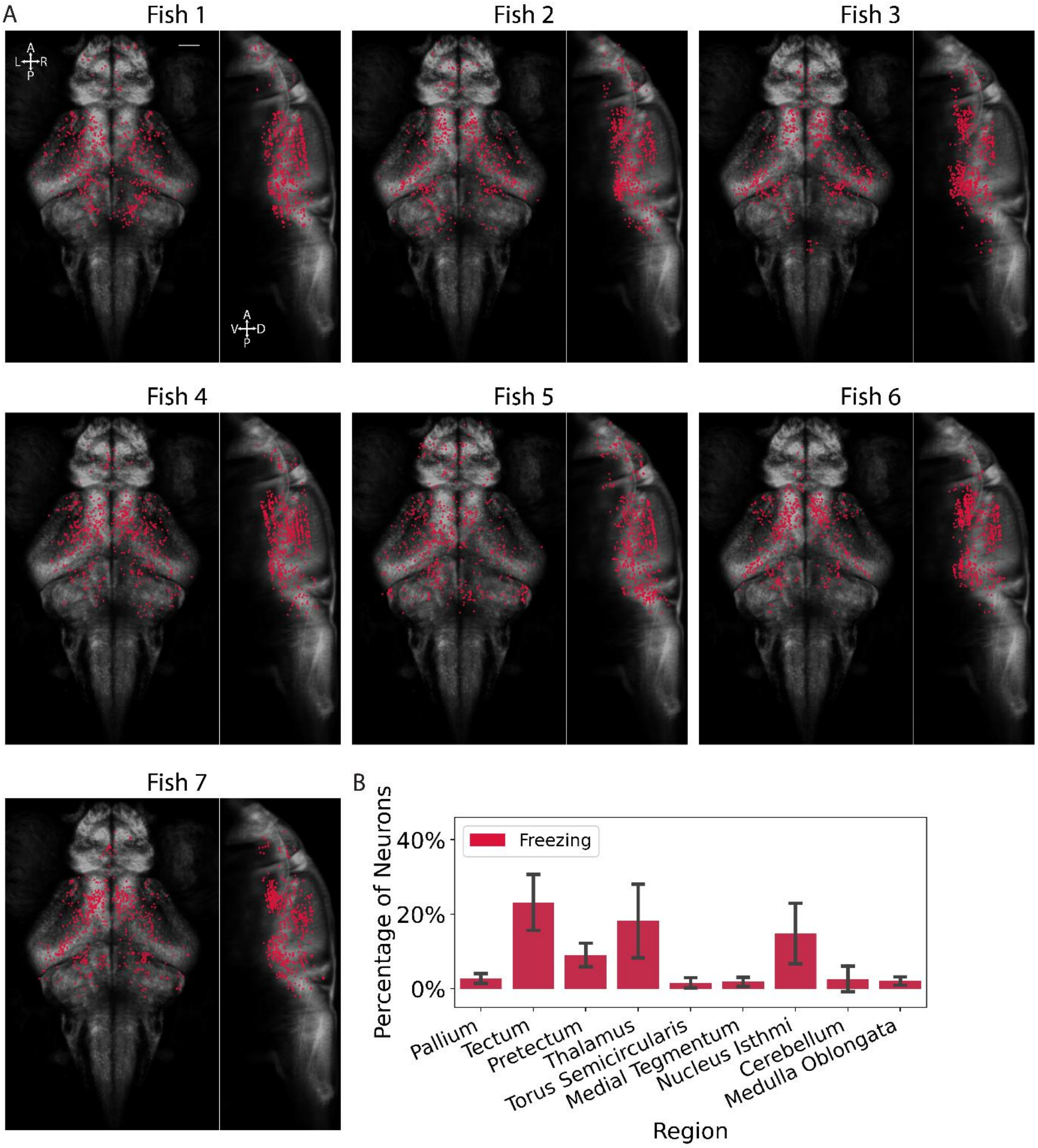

Supplementary Figure 6: Neurons with high freezing MSI are found in the same anatomical regions across seven larvae. (A) Anatomical locations of the top 600 neurons by freezing MSI in each fish, without spatial colocalization filtering. (B) Percentage of the top 600 freezing MSI neurons in each area across seven fish. Error bar represents Standard Deviation.

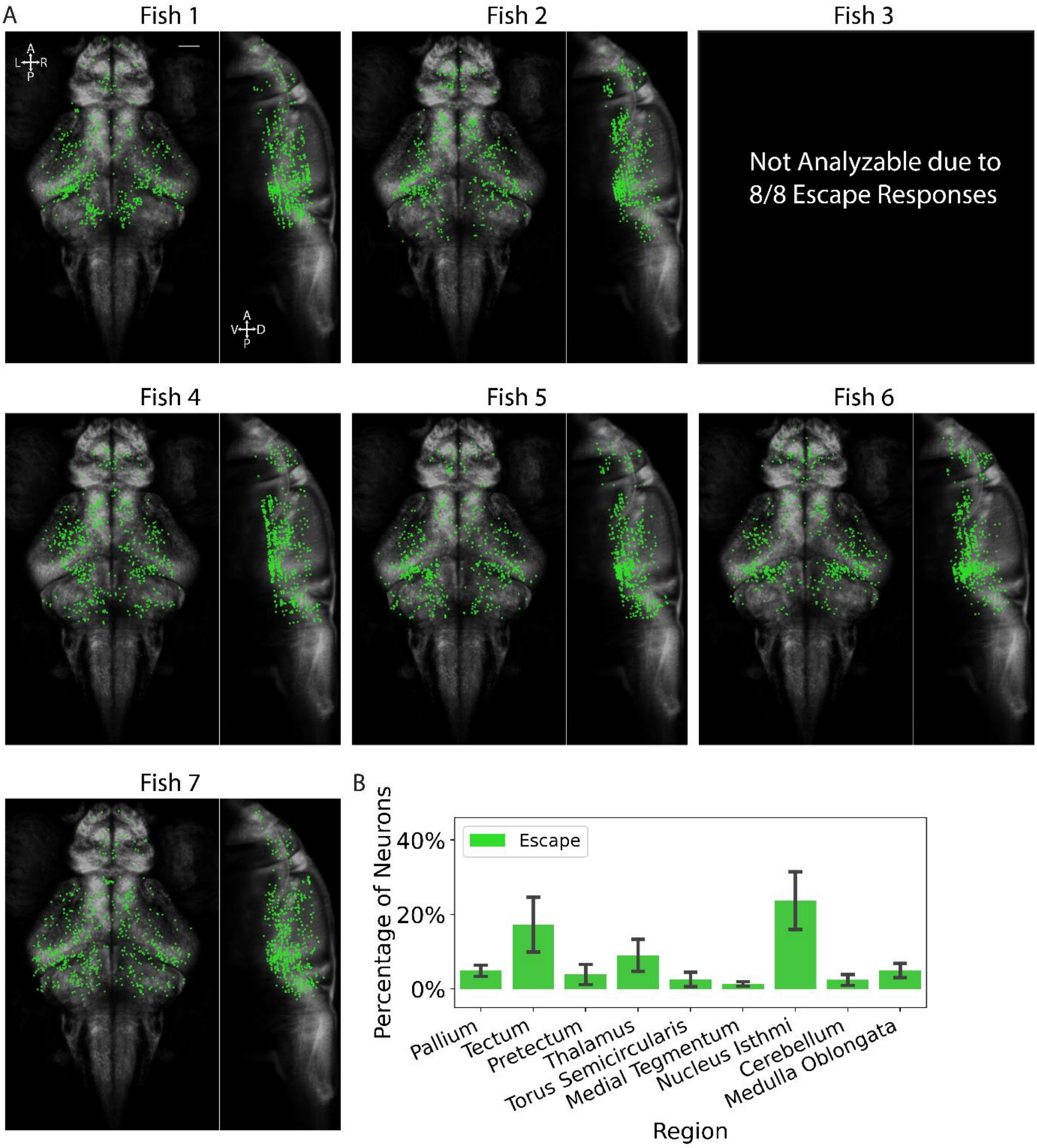

Supplementary Figure 7: Neurons with high escape MSI are found in the same anatomical regions across six larvae. (A) Anatomical locations of the top 600 neurons by escape MSI in each fish, without spatial colocalization filtering. (B) Percentage of top 600 escape MSI neurons in each area across seven fish. Error bar represents Standard Deviation.

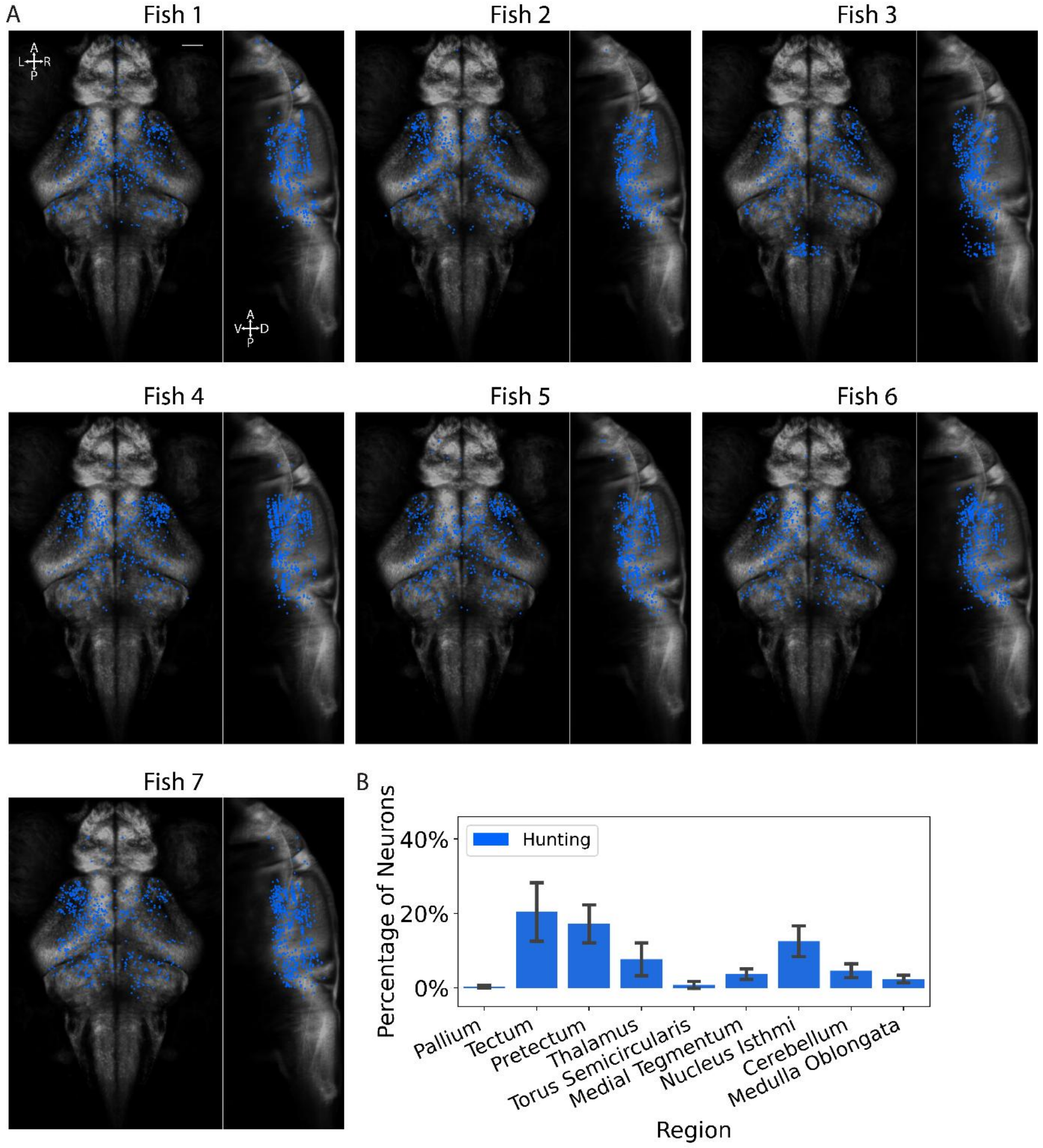

Supplementary Figure 8: Neurons with high hunting MSI are found in the same anatomical regions across seven larvae. (A) Anatomical locations of the top 600 neurons by hunting MSI in each fish, without spatial colocalization filtering. (B) Percentage of the top 600 hunting MSI neurons in each area across seven fish. Error bar represents Standard Deviation.

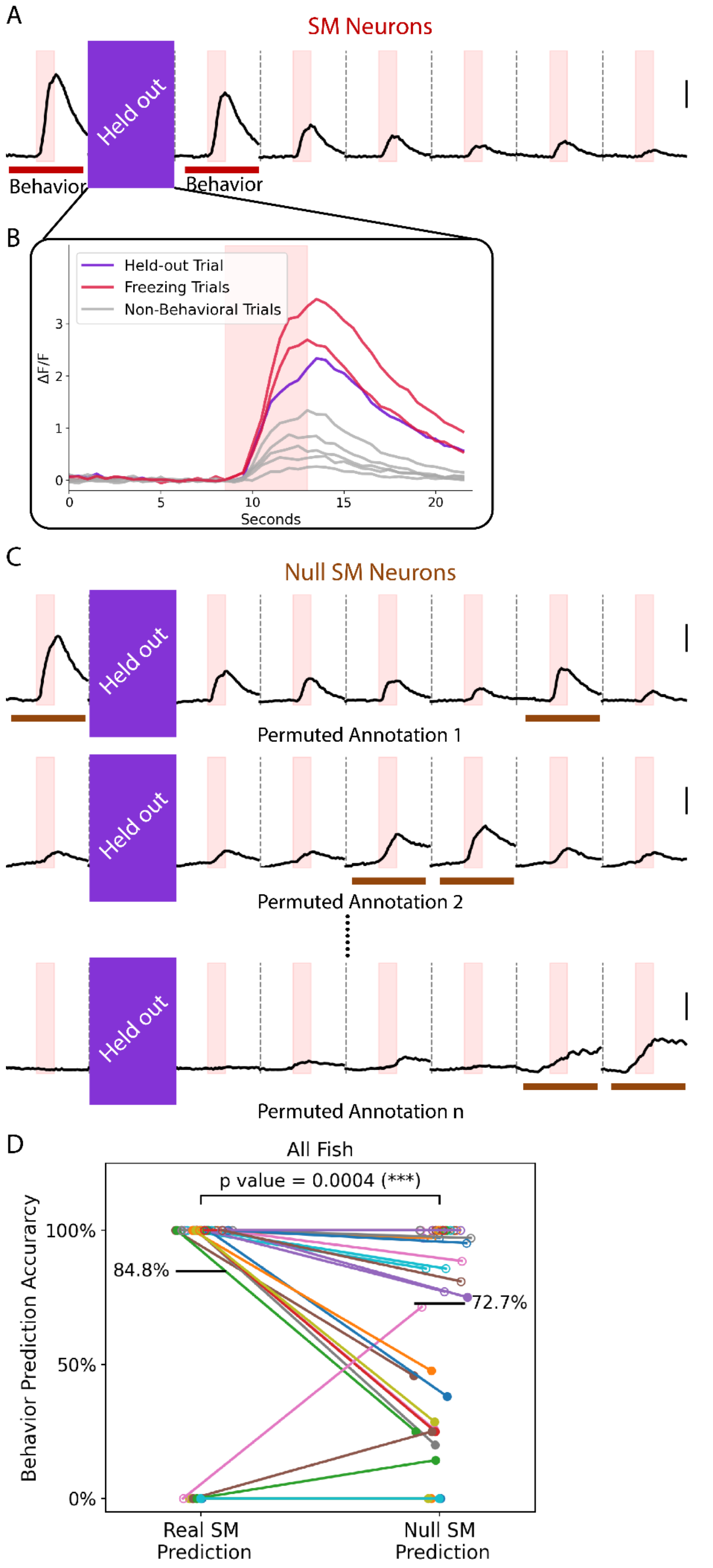

Supplementary Figure 9: Freezing SM neurons can predict freezing behavior. (A) Average response of the freezing SM neurons in one fish, selected based on activity in 7 trials, with the second trial held out. Scale bar represents ΔF/F = 1. (B) Activity during the held-out trial (purple) compared to behavior (red) and no response trials (grey). The maximum value of the held-out trace was compared to the same value for behavior and non-behavior trials to make the prediction. (C) Average activity of the null SM neurons selected based on different permutations of the freezing annotation. Brown bars represent the freezing trials for each permutation. (D) Accuracy with which SM neurons predict the behavioral annotation (freezing or no response) of the held out trial. For null SM neurons, the ability to predict the permuted annotation (brown bars) was tested. Each dot represents one held out trial in one fish. Solid dots represent freezing trials, and open dots represent no response trials. For the null SM neurons, the dots represent their accuracy over all possible permutations of freezing annotation with a given trial held out. ***, $p<0.001$, one sided Wilcoxon signed-rank test.

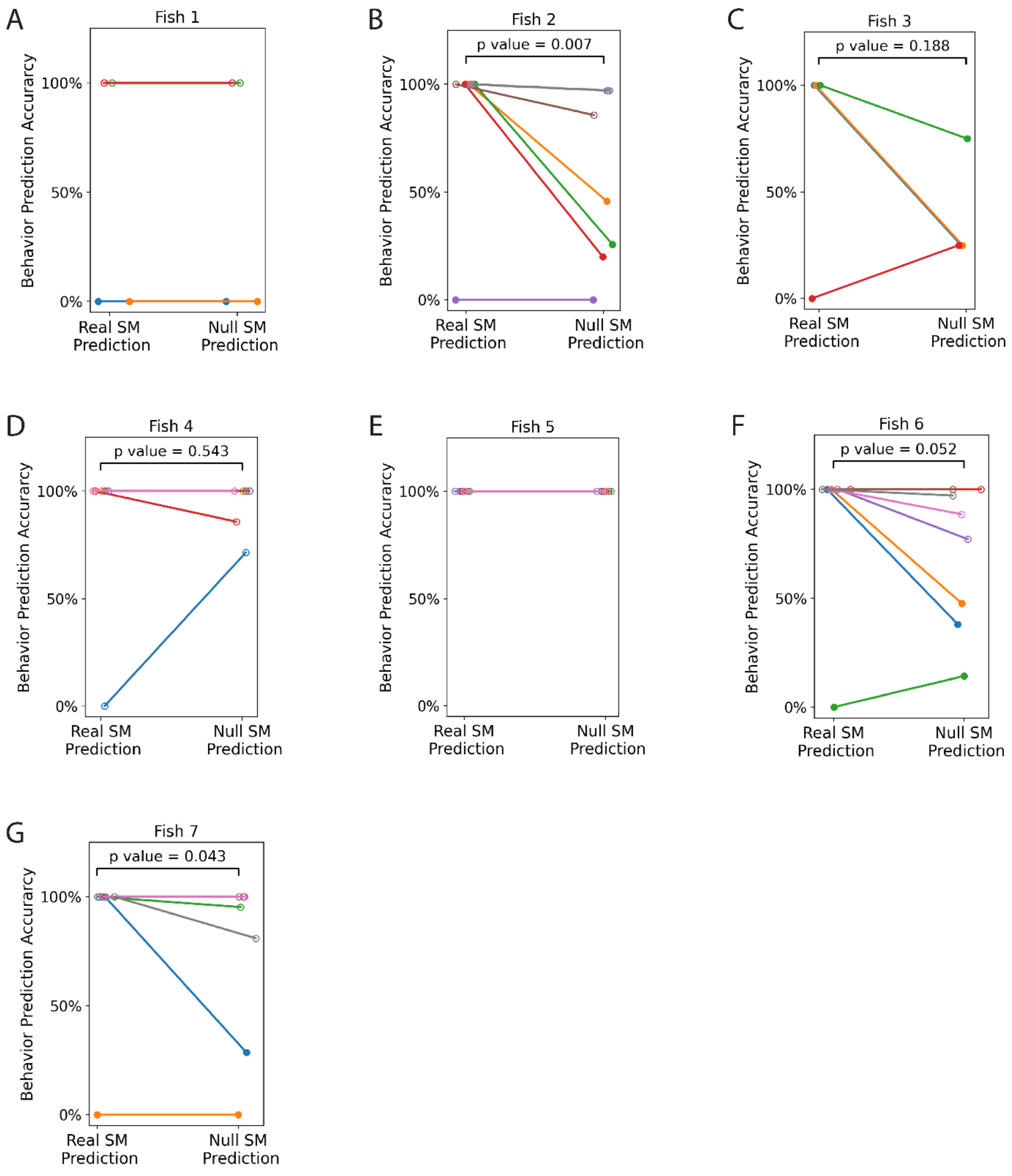

Supplementary Figure 10: Behavioral prediction accuracy of real and null SM neurons in each fish. (A-G) Accuracy with which SM neurons predict the behavioral annotation (freezing or no response) of the held out trial, as in Supplementary figure 9, for each fish. Each dot represents one held out trial in one fish. Solid dots represent freezing trials, and open dots represent no response trials. P value is calculated with the one sided Wilcoxon signed-rank test.

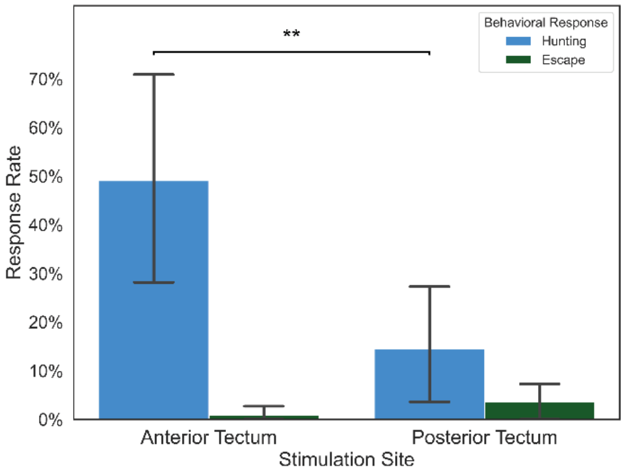

Supplementary Figure 11: Hunting and escape responses triggered by optogenetic stimulation of Anterior or Posterior tectum. Response rate is the percentage of trials annotated as having at least one episode of the behavior. Error bars represent Standard Deviation. n = 11 larvae. **, $p<0.01$. Wilcoxon Signed Rank test, one sided.

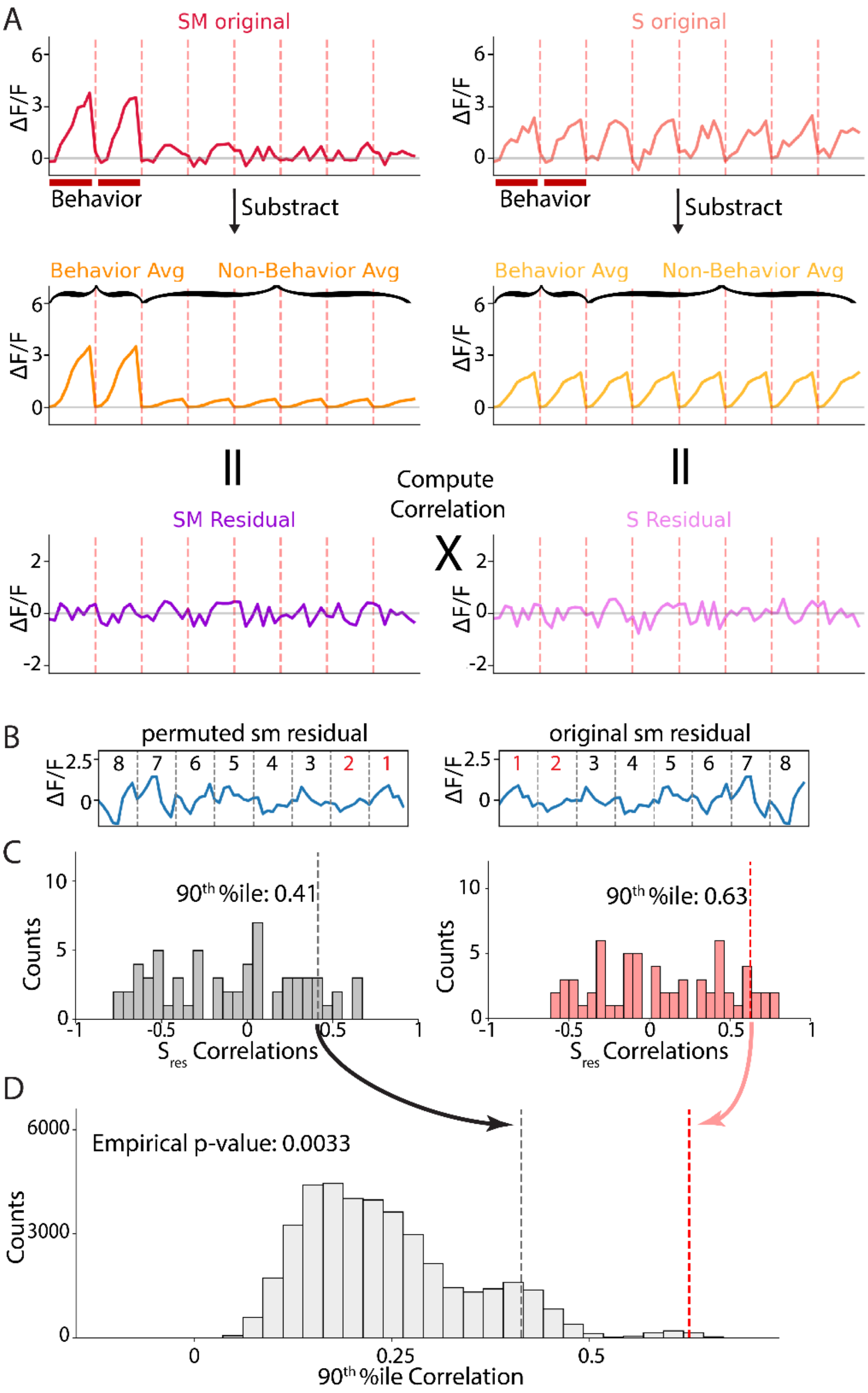

Supplementary Figure 12: Calculation of p values for SM and S partial correlations. (A) Calculation of SM and S residual traces and their correlation ρ(S,SM│B), i.e., the partial correlation, for a schematic freezing SM and sweep sensory neuron. (B) Example of trial-permuted and original SM residual traces for one freezing SM neuron. (C) Grey histogram: distribution of correlations between the permuted SM residual and the sweep sensory neurons in the same tectum. Pink histogram: distribution of correlations between the original SM residual and the sweep sensory neurons in the same tectum. Number of datapoints = Number of tectal sweep sensory neurons for that fish. (D) Grey dotted line: 90th%ile of one example permuted SM_{res} to S_{res} correlations. Light grey histogram: the distribution of the aforementioned 90th%ile's across the 8!=40,320 permutations of the SM residual. Red dotted line: 90th%ile of the original S_{res} correlation with sensory residuals. Empirical p-value was calculated as the number of permutations whose 90th%ile correlation value was equal or greater than the original SM_{res} 90th%ile correlation value (red dotted line) divided by the total number of permutations.

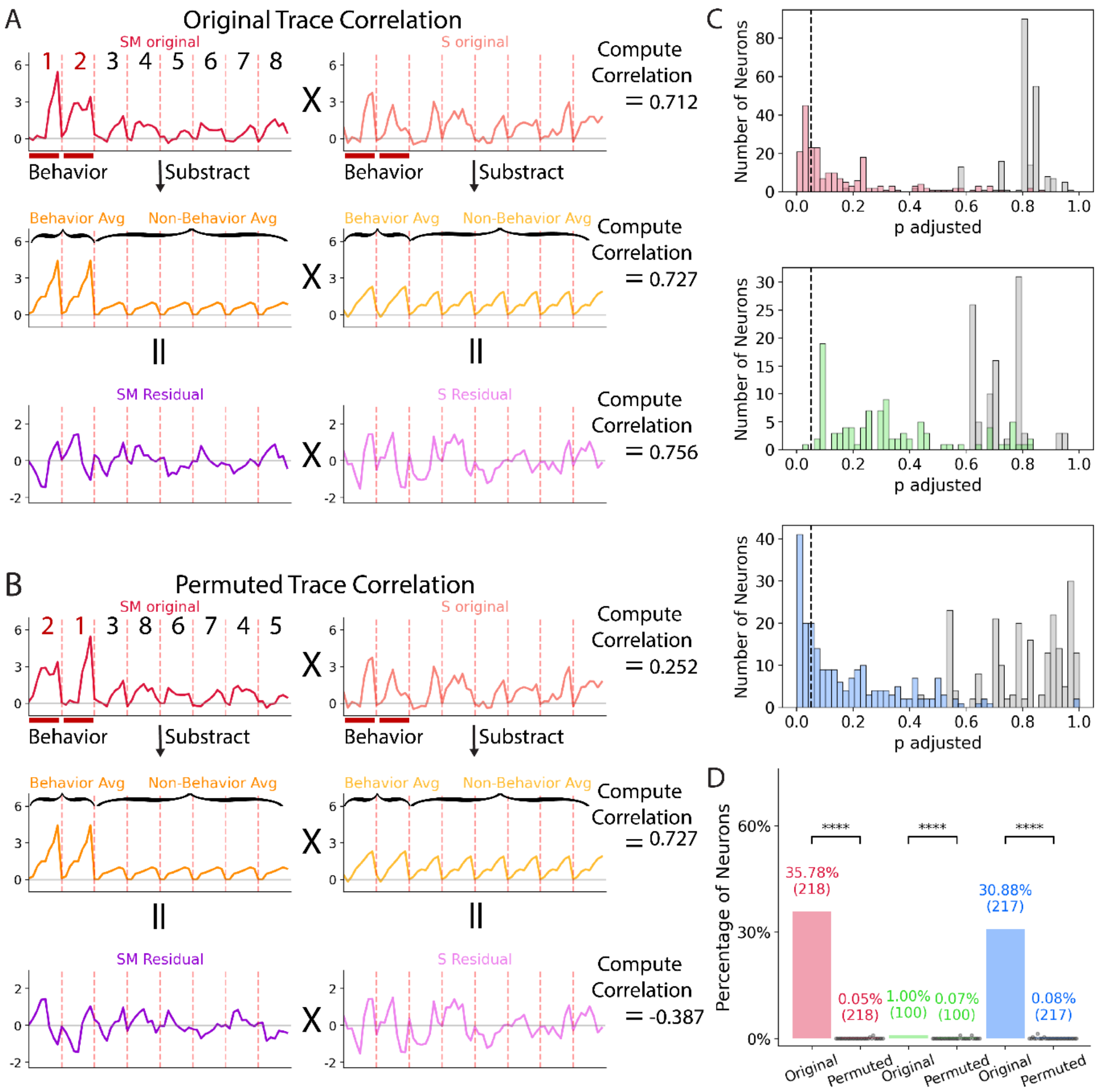

Supplementary Figure 13: Subtraction of average traces does not create the correlation residuals. (A) Calculation of SM to S residual correlation for a pair of example neurons from our dataset. (B) Calculation of the correlation between the S residual and the SM residual after the SM neuron's trace was permuted within behavior and non-behavior trials, leaving the behavior and non-behavior averages (middle row) unchanged. (C) Adjusted p-values for all freezing (red), escape (green), and hunting (blue) SM neurons, calculated by applying the Benjamini-Hochberg procedure to the empirical p-value from Supplementary figure 12, to adjust for testing all SM neurons combined, from all fish. Grey bars represent the adjusted p-values for SM neurons for one within category permutation as in B. Dashed lines indicate 0.05. (D) Percentage of each class of SM neuron that had a significant correlation with the corresponding S population in the original and permuted cases. In parenthesis is the total number of SM neurons of that type across all fish. Each black dot represents one of the 30 permutations sampled. ****, p<0.0001, One sided Wilcoxon signed-rank test.

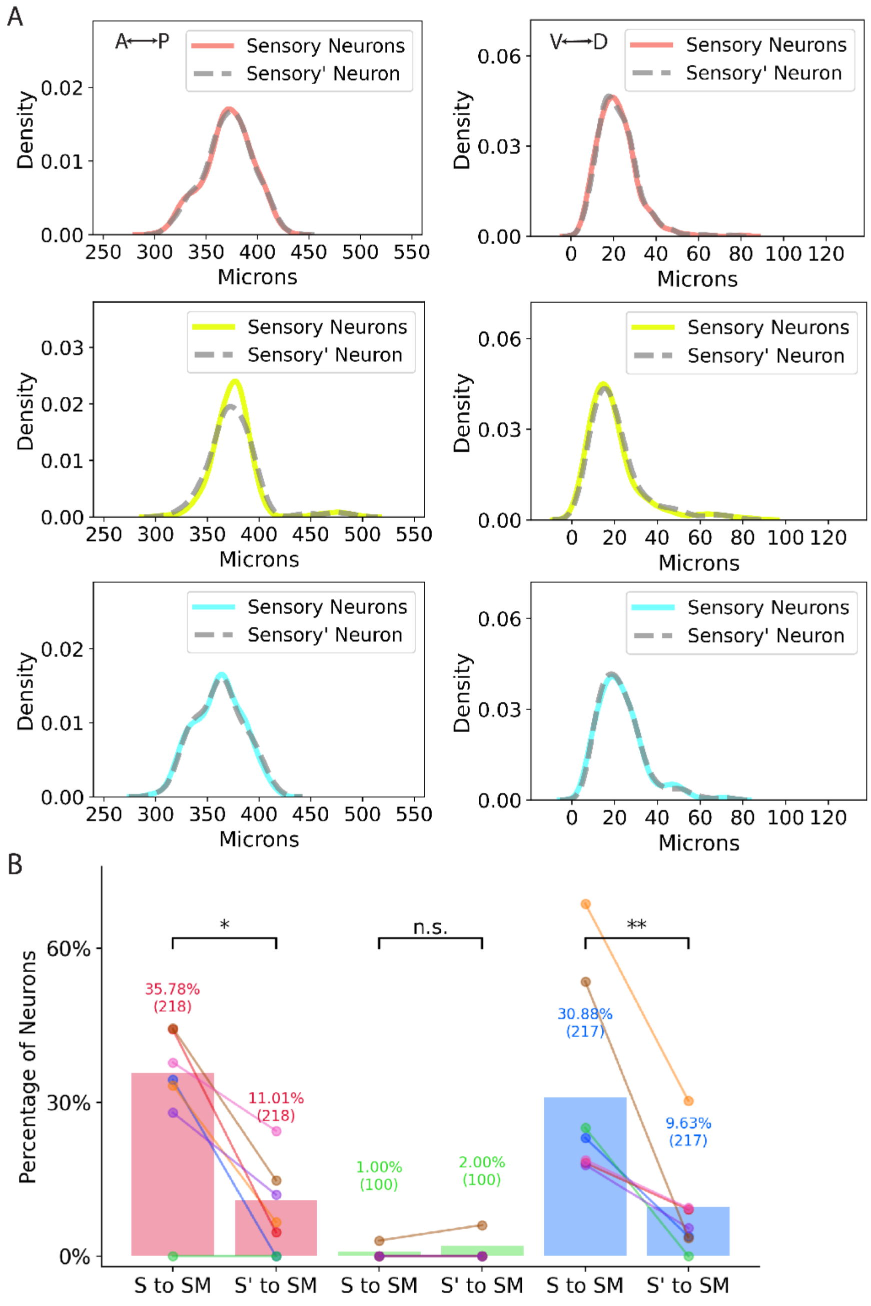

Supplementary Figure 14: Anatomically matched surrogate S neurons with average SI indices are less correlated with SM neurons than the real sensory neurons. (A) Location along the A/P and D/V axes of the tectum for sweep (red), looming (yellow-green), and prey (blue) sensory neurons and their S' counterparts (grey dashed lines). For each S neuron, an S' neuron was selected as the closest neuron in the same imaging plane with an SI +/-0.05 the average SI for tectal neurons in that fish (~.5) (B) Percentage of SM neurons that had significant correlations with S or S' neurons in the same tectum. In parenthesis is the total number of SM neurons of that type across all fish. Colored dots indicate the percentage of significant SM neurons in one fish. *, $p<0.05$; **, $p<0.01$, One sided Wilcoxon signed-rank test.

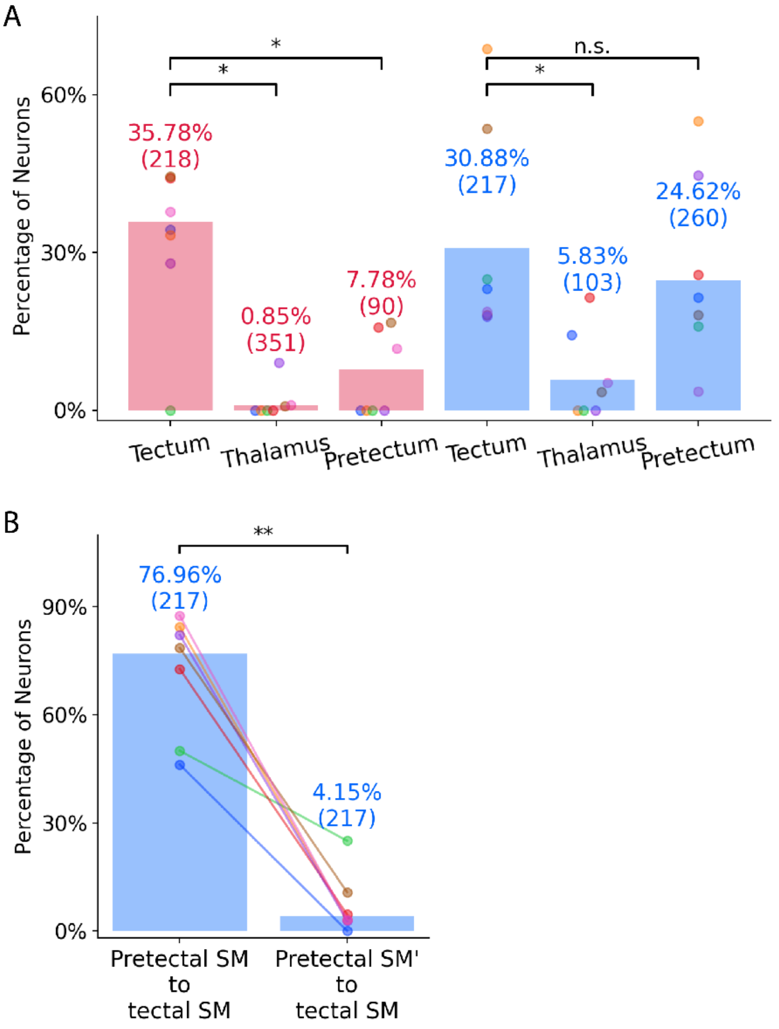

Supplementary figure 15: Partial correlations in the tectum, pretectum, and thalamus. (A) Bars represent the percentage of freezing SM (red) and hunting SM (blue) neurons in each area with a significant partial correlation with the S neurons in the same area. Each colored dot represents the number of significantly correlated SM neurons in that area in one fish. In parenthesis is the total number of SM neurons of that type across all fish. (B) Percentage of pretectal hunting SM neurons and SM' neurons with significant correlations to the tectal hunting SM population. For each pretectal SM neuron, an SM' neuron in the same imaging plane with an MSI +/-0.05 the average pretectal MSI for that fish was selected. *, $p<0.05$; **, $p<0.01$, One sided Wilcoxon signed-rank test.

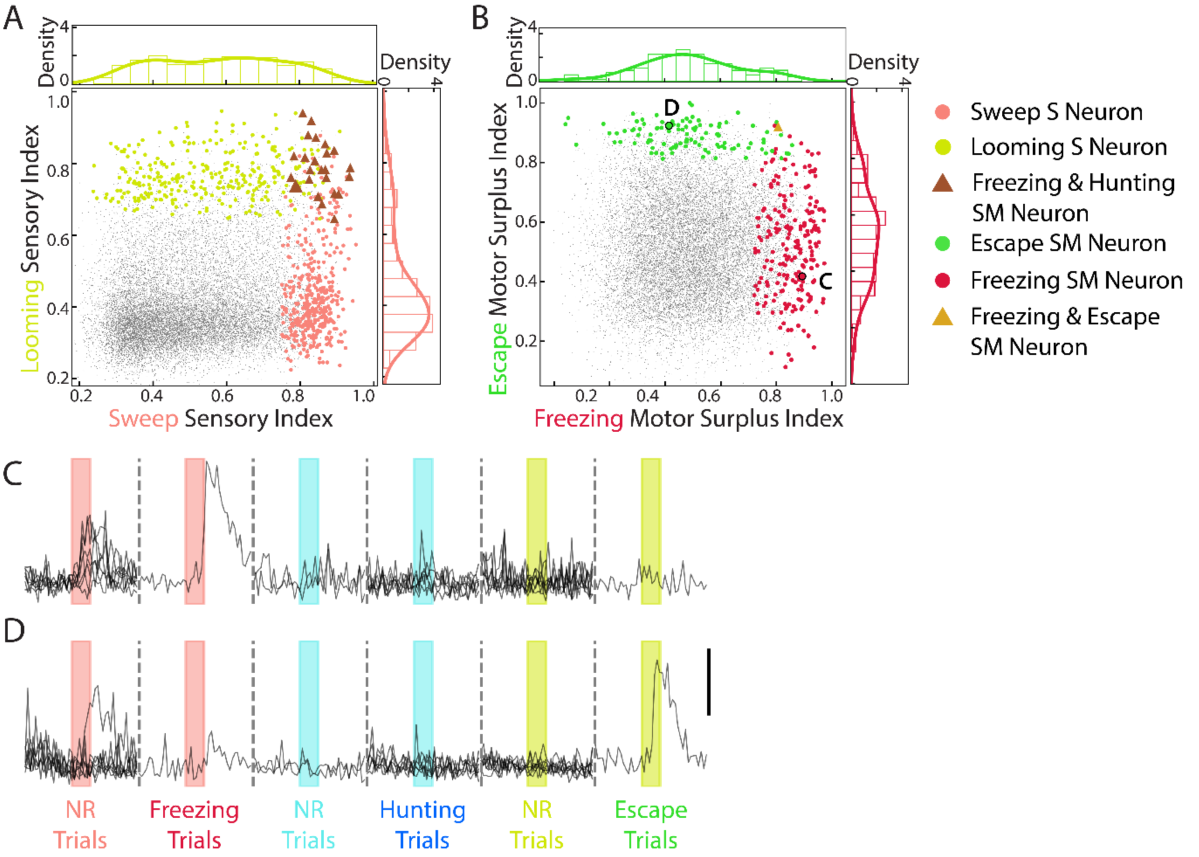

Supplementary Figure 16: Functional segregation of defensive visual pathways in the tectum. (A) The distribution of looming sensory neurons (light green) and sweep sensory neurons (light red) in the tectum on the looming and sweep SI. Crimson triangles: neurons belonging to both populations. (B) The distribution of escape (dark green) and freezing (dark red) SM neurons.Gold triangle = neuron belonging to both pobulations. (C-D) Responses of example freezing and escape SM neurons. Red, blue and green bars represent 4-second presentation of sweep, prey, and looming stimuli. Scale bar indicates ΔF/F = 3. The example neurons are noted in panel B.

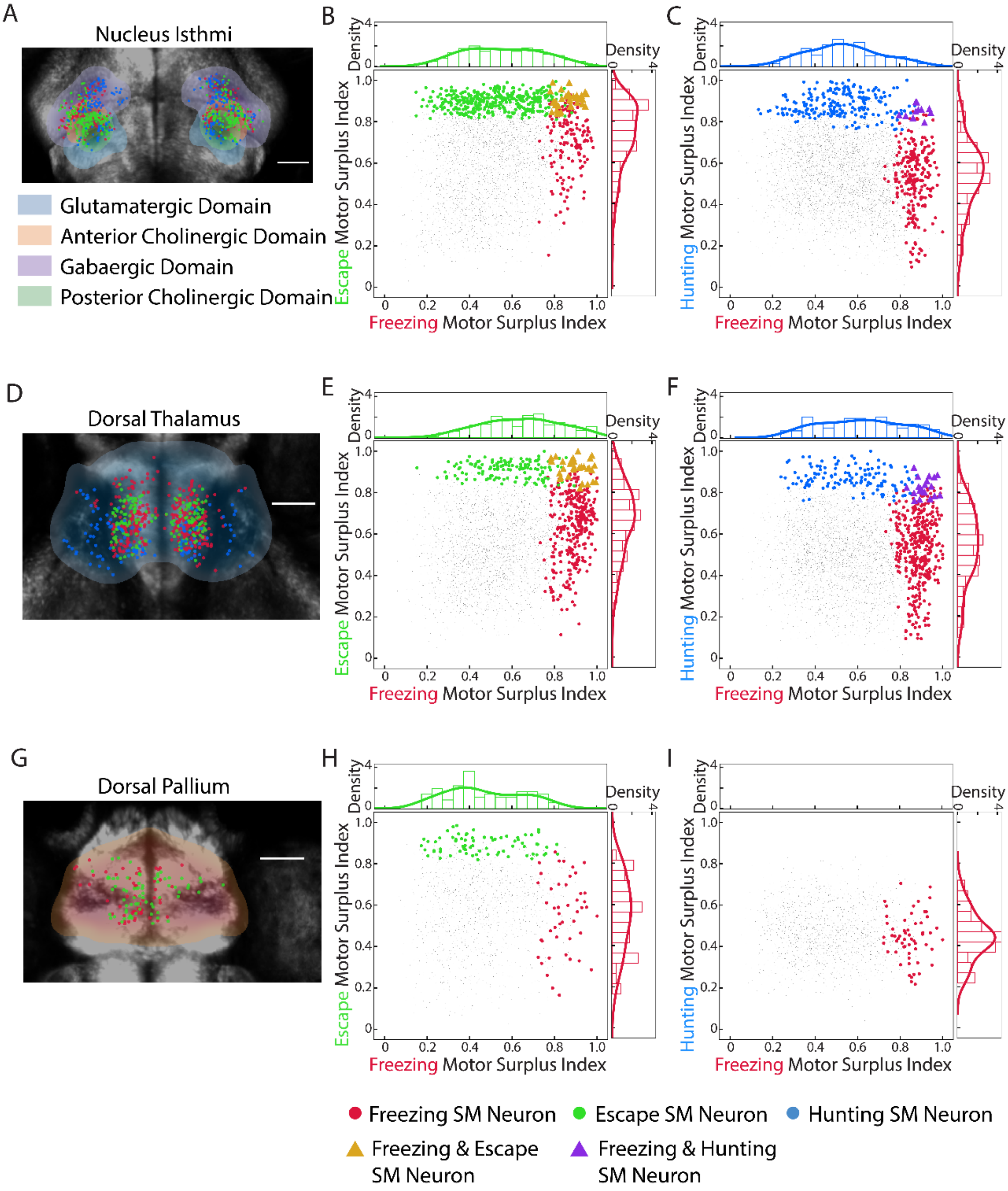

Supplementary Figure 17: Locations and functional properties of SM neurons in NI, thalamus, and pallium. (A) Dorsal view of the locations of freezing (red), escape (green), and prey (blue) SM neurons in the NI. Shaded regions indicate locations of different domains in the mapZebrain atlas. Scale bar indicates 50μm. (B) The distribution of the NI freezing (red) and escape (green) SM neurons on the MSI of freezing and escape. Golden triangles: neurons belonging to both populations. (C) The distribution of NI freezing (red) and hunting (blue) SM neurons on the MSI of freezing and hunting. Purple triangles: neurons belonging to both populations. (D) Dorsal view of the locations of the SM neurons in the thalamus. (E)

Distribution of thalamic freezing and escape SM neurons. (F) Distribution of thalamic freezing and hunting SM neurons. (G) Dorsal view of the locations of the SM neurons in the pallium. (H) Distribution of pallial freezing and ecape SM neurons. (I) Distribution of pallial freezing SM neurons (no hunting SM neurons were identified in the pallium).

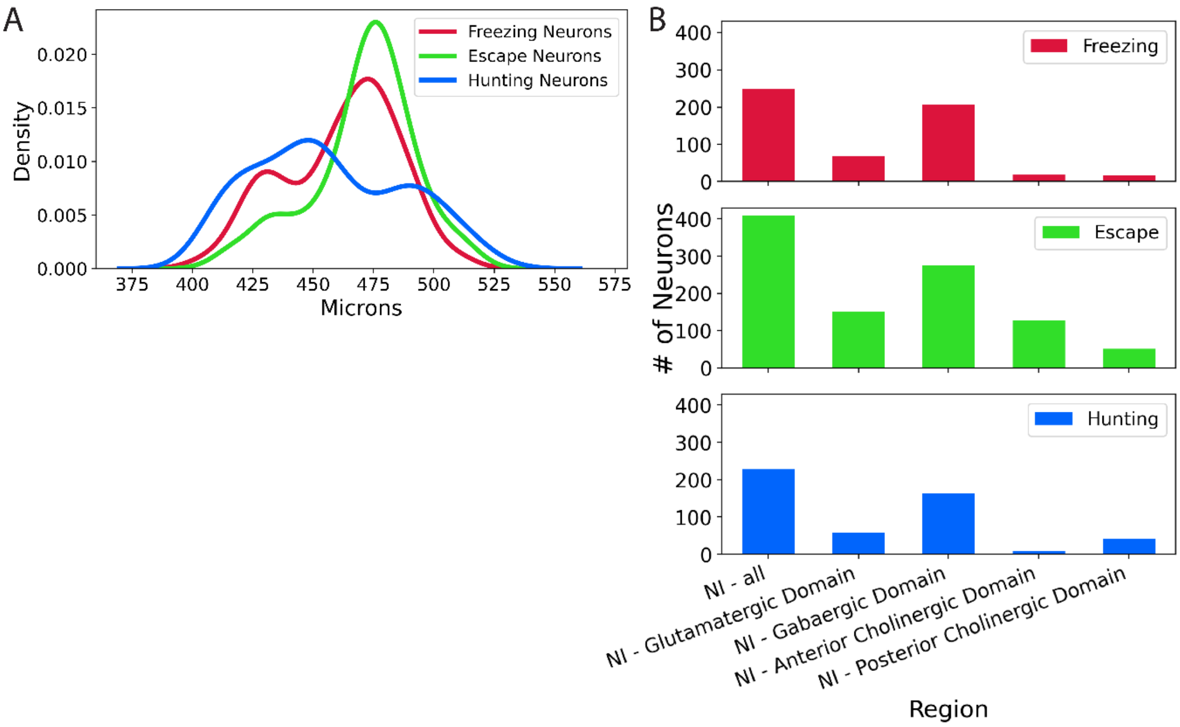

Supplementary Figure 18: Anatomical distribution of SM neurons in the nucleus isthmi, and functional properties of all SM neurons. (A) Distribution of hunting, freezing and escape SM neurons in the nucleus isthmi along the anterior to posterior axis. (B) Numbers of SM neurons total and in each subregion of the nucleus isthmi. (C) Average activity of SM neurons in the whole imaging volume during behavior or no response trials. Pink bars respresent 4-second stimulus presentation. n = 7 larvae.

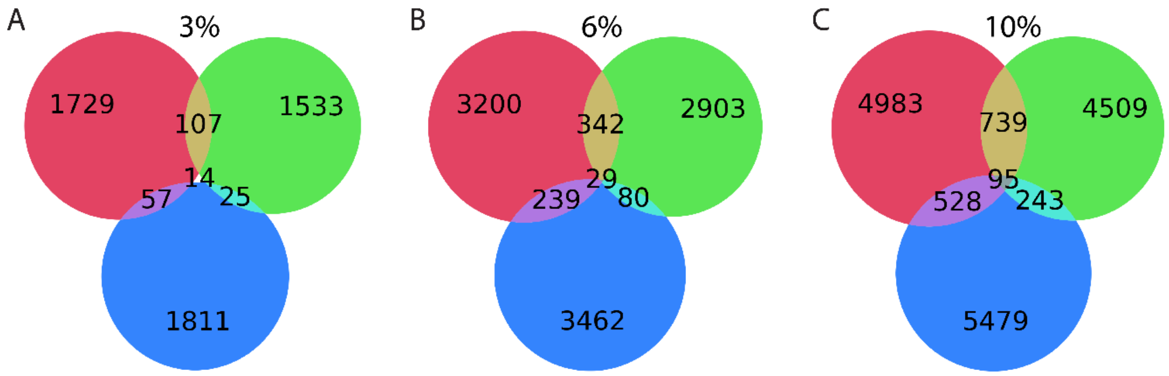

Supplementary Figure 19: Functional properties of all SM neurons. (A) Venn diagram showing the overlap among freezing (red), escape (green), and hunting (blue) SM populations using 3% as the threshiold. (B) Overlap when 6% is taken as the SM neuron threshold. (C) Overlap when 10% is taken as the SM neuron threshold.

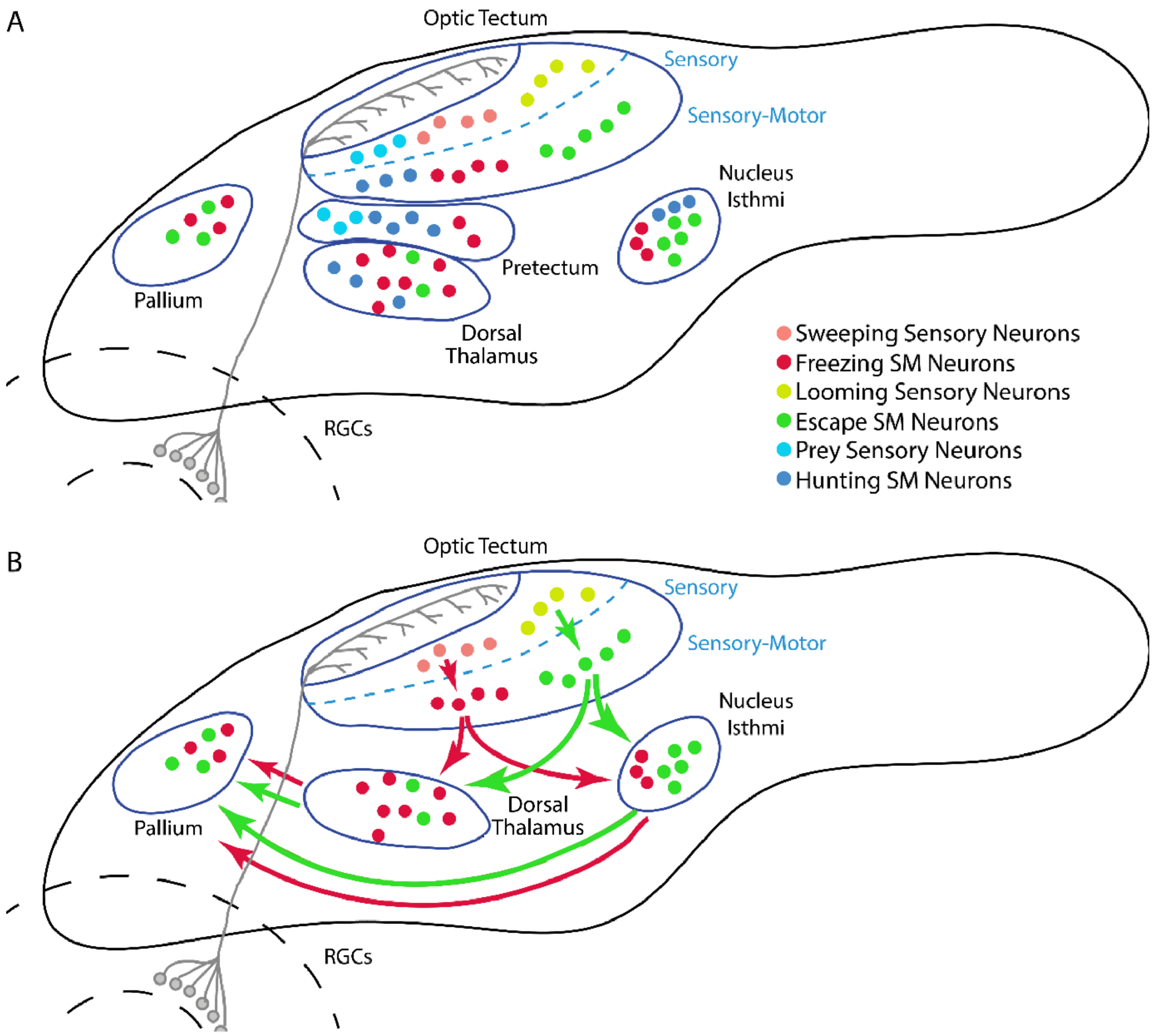

Supplementary Figure 20: Sensory motor transformation in the visual system of larval zebrafish. (A) Schematic of the locations of sensory and sensory-motor (SM) neurons in the brain. (B) Proposed defensive behavior pathways based on homogous areas in rodents.

Supplementary Table 1: Number of Segmented Neurons in Each Brain Region

Region	Number of Neurons Segmented
All forebrain areas	47,048
Telencephalon	25,273
Olfactory Bulb	3,615
Pallium (dorsal telencephalon)	18,498
Subpallium (ventral telencephalon)	3,160
Eminentia Thalami	1,549
Prethalamus (alar prosomere 3, ventral thalamus)	1,190
Epiphysis	78
Habenula	2,840
Dorsal Habenula	960
Ventral Habenula	1,880
Thalamus Proper	8,970
Pretectum	13,071
Posterior Tuberculum (basal part of prethalamus and thalamus)	276
All midbrain areas	53,160
Tegmentum	7,926
Tectum	40,824
Periventricular layer	32,886
Tectal Neuropil	7,843
Torus Longitudinalis	342
Torus Semicircularis	3,969
All hindbrain areas	54,036
Cerebellum	12,862
Medulla oblongata	41,174
Superior Medulla Oblongata	32,250
Superior Dorsal Medulla Oblongata	18,521
Superior Ventral Medulla Oblongata	13,726
Anterior (dorsal) trigeminal motor nucleus	977
Posterior (dorsal) trigeminal motor nucleus	113
Nucleus Isthmi	10,277
Anterior cholinergic domain of the Nucleus Isthmi	1,746
Posterior cholinergic domain of the Nucleus Isthmi	1,258

Glutamatergic domain of the Nucleus Isthmi	3,547
Gabanergic domain of the Nucleus Isthmi	6,287
Superior Ventral Medulla Oblongata (remaining)	1,494
Locus Coeruleus	246
Interpeduncular nucleus	38
Superior Raphe	584
Intermediate Medulla Oblongata	8,733
Inferior Medulla Oblongata	63

Supplementary Video Legends

Supplementary Video 1: Sweep stimulus suppresses spontaneous swims. Swimming was recorded from above at 200 frames per second. The stimulus was a 15° diameter dark disk moving at 60°/second on a red background.

Supplementary Video 2: Sweep stimulus causes a reduction in heart rate. The stimulus was a 15° diameter dark disk moving at 60°/second on a red background. Heart rate was recorded from the side at 100 frames per second.

Supplementary Video 3: Prey stimulus triggers hunting behavior. Eye and tail movements recorded from above at 200 frames per second, in response to a 4° diameter UV dot moving at 120°/second for 6 seconds. For imaging experiments, the speed was 60°/second and duration was 4 seconds.

Supplementary Video 4: Looming stimulus triggers escape behavior. Tail movements were recorded from above at 200 frames per second, in response to a dark disk expanding to 60° in diameter.

SI References

1. Khan, B., Jaesiri, O., Lazarte, I.P., Li, Y., Tian, G., Zhao, P., Zhao, Y., Ho, V.D., and Semmelhack, J.L. (2023). Zebrafish larvae use stimulus intensity and contrast to estimate distance to prey. Curr. Biol. https://doi.org/10.1016/j.cub.2023.06.046.

2. Khan, B., Lazarte, I.P., Jaesiri, O., Zhao, P., and Semmelhack, J.L. (2024). Protocol for using UV stimuli to evoke prey capture strikes in head-fixed zebrafish larvae. STAR Protoc. *5*, 102780. https://doi.org/10.1016/j.xpro.2023.102780.

3. Signoret-Genest, J., Schukraft, N., L. Reis, S., Segebarth, D., Deisseroth, K., and Tovote, P. (2023). Integrated cardio-behavioral responses to threat define defensive states. Nat. Neurosci. *26*, 447–457. https://doi.org/10.1038/s41593-022-01252-w.

4. Pachitariu, M., Stringer, C., Dipoppa, M., Schröder, S., Rossi, L.F., Dalgleish, H., Carandini, M., and Harris, K.D. (2017). Suite2p: beyond 10,000 neurons with standard two-photon microscopy. bioRxiv, 061507. https://doi.org/10.1101/061507.

5. Kunst, M., Laurell, E., Mokayes, N., Kramer, A., Kubo, F., Fernandes, A.M., Förster, D., Dal Maschio, M., and Baier, H. (2019). A Cellular-Resolution Atlas of the Larval Zebrafish Brain. Neuron *103*, 21-38.e5. https://doi.org/10.1016/j.neuron.2019.04.034.

6. Dana, H., Sun, Y., Mohar, B., Hulse, B.K., Kerlin, A.M., Hasseman, J.P., Tsegaye, G., Tsang, A., Wong, A., Patel, R., et al. (2019). High-performance calcium sensors for imaging activity in neuronal populations and microcompartments. Nat. Methods *16*, 649–657. https://doi.org/10.1038/s41592-019-0435-6.

7. Chen, X., Mu, Y., Hu, Y., Kuan, A.T., Nikitchenko, M., Randlett, O., Chen, A.B., Gavornik, J.P., Sompolinsky, H., Engert, F., et al. (2018). Brain-wide Organization of Neuronal Activity and Convergent Sensorimotor Transformations in Larval Zebrafish. Neuron *100*, 876-890.e5. https://doi.org/10.1016/j.neuron.2018.09.042.

8. Marques, J.C., Li, M., Schaak, D., Robson, D.N., and Li, J.M. (2020). Internal state dynamics shape brainwide activity and foraging behaviour. Nature *577*, 239–243. https://doi.org/10.1038/s41586-019-1858-z.

9. Pearl, J. (2009). Causality 2nd ed. (Cambridge University Press) https://doi.org/10.1017/CBO9780511803161.
